# Path planning for autonomous mobile robots using multi-objective evolutionary particle swarm optimization

**DOI:** 10.1371/journal.pone.0271924

**Published:** 2022-08-19

**Authors:** Ittikon Thammachantuek, Mahasak Ketcham

**Affiliations:** 1 Department of Information Technology, Faculty of Information Technology and Digital Innovation, King Mongkut’s University of Technology North Bangkok, Bangkok, Thailand; 2 Department of Information Technology Management, Faculty of Information Technology and Digital Innovation, King Mongkut’s University of Technology North Bangkok, Bangkok, Thailand; Torrens University Australia, AUSTRALIA

## Abstract

In this article, a new path planning algorithm is proposed. The algorithm is developed on the basis of the algorithm for finding the best value using multi-objective evolutionary particle swarm optimization, known as the MOEPSO. The proposed algorithm is used for the path planning of autonomous mobile robots in both static and dynamic environments. The paths must follow the determined criteria, namely, the shortest path, the smoothest path, and the safest path. In addition, the algorithm considers the degree of mutation, crossover, and selection to improve the efficiency of each particle. Furthermore, a weight adjustment method is proposed for the movement of particles in each iteration to increase the chance of finding the best fit solution. In addition, a method to manage feasible waypoints within the radius of obstacles or blocked by obstacles is proposed using a simple random method. The main contribution of this article is the development of a new path planning algorithm for autonomous mobile robots. This algorithm can build the shortest, smoothest, and safest paths for robots. It also offers an evolutionary operator to prevent falling into a local optimum. The proposed algorithm uses path finding simulation in a static environment and dynamic environment in conjunction with comparing performance to path planning algorithms in previous studies. In the static environment (4 obstacles), the shortest path obtained from the proposed algorithm is 14.3222 m. In the static environment (5 obstacles), the shortest path obtained from the proposed algorithm is 14.5989 m. In the static environment (6 obstacles), the shortest path obtained from the proposed algorithm is 14.4743 m. In the dynamic environment the shortest path is 12.2381 m. The results show that the proposed algorithm can determine the paths from the starting point to the destination with the shortest distances that require the shortest processing time.

## Introduction

Currently, autonomous vehicles and autonomous mobile robots are in wide use. They are used to deliver goods from sellers to buyers and to deliver goods within warehouses. In factories, they are used to carry goods to conveyor belts. In addition, they are required to work in dangerous areas such as military and mining operations. Autonomous mobile robots can reach destinations safely according to work objectives. Therefore, fast and accurate path planning is a significant factor. Path planning is an important process for autonomous mobile robots, as this helps robots move from a starting point to a destination without hitting any obstacles. Generally, path planning for autonomous mobile robots is divided into two categories: global path planning and local path planning. Global path planning is used when robots have environmental information, including obstacles and goals of traveling. In contrast, local path planning is for robots that do not have information about the environment while traveling. Meanwhile, the environment can change at all times [1].

Path planning solutions for autonomous mobile robots can be divided into two methods: in classic methods, such as the cell decomposition method [2, 3], the potential field method [4–7], the subgoal method [8, 9], path planning can find required paths but requires a long time to process information [10, 11]. Reactive methods can also be used for the path planning of robots. In general, reactive methods require a metaheuristic algorithm to process information. This helps build paths in environments that seem to change frequently. Recently, many methods have been developed to solve path planning problems for autonomous mobile robots. These methods can be divided into 5 groups [12]. The first group is population-based algorithms/nature-inspired algorithms. They are used to plan the best fit paths for autonomous mobile robots. These methods can solve problems stuck in local optimums. However, this requires long processing times. The second group is heuristic algorithms. These methods are used to find the best fit paths, similar to the first group. The major restriction of these methods is that they must be conducted in static environments, and only the distance criterion can be used to evaluate path suitability. The third group is artificial neural networks. These methods are used to find paths in real time, and a multilayer perceptron is the most common class found in this group. However, they can take a long time to prepare information examples and teach robots to be intelligent. The fourth group is fuzzy logic algorithms. These methods stimulate the way human brains work in finding paths. They are typically used to manage static environments. The last group is hybrid algorithms. In these methods, two heuristic algorithms work together to find optimal paths. The major restriction of these methods is the complexity of the algorithms, as two or more algorithms must work together. Based on recent studies [12], problems of autonomous mobile robot path planning using the aforesaid methods were concluded in three aspects as they use a lot of resources to figure out an optimal path, the most common solution is a local minimum, and inaccuracy of the solution. As a consequence, a new heuristic method for autonomous mobile robot path planning was proposed by adopting an algorithm that finds the best fit value using particle swarm optimization (PSO) in conjunction with a modified bat algorithm. Though the study results showed that the proposed algorithm could find the optimal paths in static environments and dynamic environments, this algorithm could build paths in which only path length and path smoothness were taken into consideration, leading to risks that the robots could possibly collide with obstacles. Metaheuristics play an important role in solving optimization problems. The majority of such algorithms are inspired by collective intelligence and foraging of creatures in nature.

Recently, new metaheuristics have been proposed. Evolutionary particle swarm optimization (EPSO) can be classified as an optimization algorithm. It based on the sociality of bird flocks looking for food. It starts with a population of particles whose positions represent the potential solutions in search space of the studied problem. Mostly the group of particles or swarm is randomly initialized to generate the velocity and position of each particle. Each position of a particle has a fitness value which is evaluated by the fitness function to be optimized, and its velocity which controls the flying. In each iteration, all particles are updated by following two best values. The first one is the best solution it has achieved and this value is called the personal best position or pbest. Another best value is tracked by the swarm, and is that obtained so far by any particle in the population. This best value is a global best position or gbest. Moreover, three operators adopted from the differential evolution (DE): mutation, crossover, and selection are used for the smart searching. A particle’s velocity and position are updated until the convergence criterion is met, then the best particle found so far is taken as the solution [13]. Remora Optimization algorithm (ROA) can be classified as a natural-inspired and meta-heuristic algorithm. It based on the remora behavior looking for food. Remora is the name describing eight species of marine fishes in the Family Echeneidae. In the remora’s foraging process, it moves with other larger marine animals as a host. When the host reaches the sea area rich in bait, the remora will leave the host, ingest food, and then adsorb to the new host and continue to transfer to another sea area. In some large hosts, remora feeds on the parasite lived on host’s skin, for example in the case of giant whales. In some small hosts, remora follows the host to move to the bait-rich area to prey other marine animals. In the case of these two update methods, remora also makes some judges based on experience. If it takes the initiative to prey, it updates the host, makes a global update. If it eat around the host, remora does not change the host, and continues to local update. This algorithm is more inclined to provide a new idea for memetic algorithm [14]. Gorilla troops optimizer (GTO) is inspired by gorilla swarm behavior. The gorilla lives in a group called troop, composed of an adult male gorilla also known as the silverback, multiple adult female gorillas and their offspring. Moreover, the silverback is the head of the whole troop, taking all decisions, mediating disputes, directing others to food resources, determining group movements, and being responsible for safety. Younger male gorillas at the age of 8 to 12 years are called black backs since they still lack silver-color back hairs. They are affiliated with the silverback and act as backup defenders for the group. In general, both female and male gorillas tend to migrate from the group where they were born to a second new group. Alternatively, mature male gorillas are also likely to separate from their original group and constitute troops for their own by attracting migrating females. However, some male gorillas sometimes choose to stay in the initial troop and continue to follow the silverback. If the silverback dies, these males might engage in a brutal battle for dominance of the group and mating with adult females. GTO simulates different methods. Migrating to anonymous place, migrating for other gorillas, they move toward a known region, keep tracking the silverback; next, competing for mature females. They are imitated and shown to illustrate the optimization process. They are imitated and shown to illustrate the optimization process’ exploration and exploitation. Three methods are employed during the exploration stage: moving toward an anonymous region, migrating to the remaining members, and migrating toward a known region. In the exploitation stage, two tactics are used: keeping track of the silver back and then competing for mature females [15]. African vultures optimization algorithm (AVOA) is a new nature-inspired metaheuristic algorithm. It was proposed by simulating and modeling the foraging behavior and living habits of African vultures. According to the living habits of African vultures, the vultures in the population are divided into three groups. If the fitness value of the feasible solution is used to measure the quality position of the vultures, the first group is to find the best feasible solution among all vultures. The second group is that the feasible solution is the second best among all vultures. In addition to the above two vulture groups, the remaining vultures are divided into the third group. The vulture’s foraging habit is through the population together. Therefore, different types of vultures play different roles in the population. Similarly, if it is assumed that the fitness value of the feasible solution in the population can represent the advantages and disadvantages of vultures, the weakest and hungriest vultures correspond to the worst vultures at present. In contrast, the strongest and most abundant vulture corresponds to the best vulture at present. In AOVA, all vultures try to get close to he best vultures and stay away from the worst vultures [16].

In this article, a new path planning algorithm is developed to plan path finding for autonomous mobile robots. The algorithm is based on multi-objective evolutionary particle swarm optimization (MOEPSO), which uses evolutionary operators such as crossover, mutation and selection. These techniques help obtain global optimums. Moreover, the proposed algorithm can find optimal paths in terms of path length, smoothness and safety. This algorithm is comprised of three main parts. The first part builds feasible waypoints by considering path length, smoothness and safety using MOEPSO, which improves the process of weighted value adjustment for the movement of particle swarms in each iteration to fit path planning in this study. The second part offers a method to improve the feasible waypoints whose positions are located within a radius of obstacles or blocked by obstacles to allow robots to avoid obstacles they are facing. The last part proposes a method to detect obstacles using a single sensor that can detect obstacles in all directions to build obstacle-avoiding paths.

The main contribution of this article is the development of a new path planning algorithm for autonomous mobile robots. The outstanding feature of this algorithm is that it can build the shortest, smoothest, and safest paths for robots. The algorithm also offers an evolutionary operator to prevent falling into a local optimum. In addition, its processing time is quite short since it does not work with other algorithms. Moreover, it can modify its path to avoid obstacles by detecting the position of obstacles using a single sensor. The content of this article is organized as follows. The literature review describes path planning studies using different methods. The problem statement shows the requirements and criteria used for path evaluation and MOEPSO operating procedures. Path planning is described in the proposed algorithm. The detail of the experiment is shown in the experimental design. The experimental result and discussion are proposed in the result and discussion. The conclusions and future works provide a conclusion of the experimental results and the future works.

## Literature review

In recent years, many algorithms have been developed as path planning solutions for autonomous mobile robots. These algorithms can be divided into five groups. The first group includes population-based algorithms/nature-inspired algorithms. Examples of studies in this group appeared in [17, 18], in which an algorithm to find the best fit value, ant colony optimization (ACO), was used to solve path planning problems for autonomous mobile robots in static environments. In addition, improved ant colony optimization can release from the local optimum to find optimal paths for robots. Although the improved algorithm could find optimal paths for the robots, it took a long time to process information [19].

The second group includes heuristic algorithms, bat algorithms, and particle swarm optimization (PSO). In [20] the bat algorithm was modified for mobile robot path planning in static and dynamic environment. In [18], the particle swarm optimization algorithm was improved to find the optimal paths for robots. The study results indicated that the paths obtained from the improved algorithm were shorter than the paths from the PSO algorithm. The cuckoo search algorithm was used to detect gas leaks by robots, which appeared in [21]. The experiment found that the robots could detect gas leakage points. In addition, the robots were able to avoid going into areas with a high intensity of gas leakage [21]. Other algorithms, such as bacteriologic algorithms [22], intelligent immune algorithms [23], and whale optimization algorithms [24], have been used to find the optimal path for under water vehicles. The genetic algorithm is most likely used to find the shortest paths for robots, as seen in [25]. However, these algorithms were implemented in a static environment, and only path length criteria were used to evaluate path suitability.

The third group includes neural networks. A neural network is used to simulate the complicated relationships between inputs and outputs. The adoption of neural networks for the movement of robots can be divided into three types: (1) sensor data interpretation, (2) obstacle avoidance and (3) path planning [26]. Furthermore, they are used for real-time path finding, as shown in [27]. A study conducted by [28] proposed robots moving by fuzzy neural networks. In addition, a recurrent neural network (RNN) was used to make a model identify positions to learn about movement. Testing results from the simulation found that an instructed system could identify a wide and unfamiliar environment and move to the required targets. Another study was conducted by [29] using principal component analysis (PCA) to teach robots to move toward the optimal paths. Although the neural network method could build paths as needed, it required a long time to prepare information examples and teach the robots.

The fourth group includes fuzzy logic. Other than in artificial neuron networks, fuzzy logic is used to express the uncertainty of human thought. Humans can move without much thinking or calculation. The movement of robots has tried to imitate such human behavior with the help of fuzzy logic, with robots making decisions in the form of if-then rules. The movements were broken down into work and sub behaviors [30]. A study by [31] proposed the efficient obstacle avoidance mechanism in a complex environment based on the fuzzy optimized decision function. This decision function is formulated as the compact fuzzification of the two-dimensional rule over row and column of the matrix. Thus, every decision is unique and optimized.

The last group includes hybrid algorithms. In these methods, two heuristic algorithms work together to find optimal paths. The PSO algorithm is most likely used to work with other algorithms to solve path planning problems. For example, in [32], a hybrid algorithm consisting of a genetic algorithm and PSO algorithm was proposed for optimal motion planning for a dual-arm industrial robot. The other example was a PSO algorithm working with evolutionary algorithms [33]. In addition, the cuckoo search algorithm and bat algorithm were combined to find optimal paths [34]. With regard to the recent study, the PSO algorithm was combined with the bat algorithm, as shown in [12].

The major restrictions of the abovementioned studies are that robot size was not considered in movement; the robots were considered a spot-on search space, which cannot actually be used since the movement of robots must also consider the real size of the robots. Moreover, some studies stressed finding only the shortest path in a static environment, while other studies focused only on finding the shortest path in a dynamic environment without considering the smoothness of the path. A recent study [12] revealed that the path obtained from their algorithm was the shortest and smoothest path. In addition, it could work in static environments and dynamic environments. However, the algorithm did not take safety into account. These restrictions made the distance between the robots and obstacles too small, which probably led to obstacle collision while moving. Evolutionary Particle swarm Optimization (EPSO) algorithm is defined as an optimization algorithm. It based on the sociality of bird flocks looking for food like a PSO [35, 36]. The velocity and position of each bird are identified as particle and determined in each iteration. Although, PSO is an efficient algorithm with high speed convergence, the main drawback is getting trapped in a local optimal solution since loses of diversity of swarm. In EPSO, the PSO algorithm is enhanced using three operators adopted from the differential evolution (DE) algorithm for escaping from possible local optimal solution and reduces execution time. The three main efficient operators that affect the performance of the algorithm are mutation, crossover, and selection. We will describe their processes and mechanisms for path planning in the next section.

The main contribution of this article is the development of a new path planning algorithm for autonomous mobile robots. The outstanding feature of this algorithm is that it can build the shortest, smoothest, and safest paths for robots. The algorithm also offers an evolutionary operator to prevent falling into a local optimum. In addition, its processing time is quite short since it does not work with other algorithms. Moreover, it can modify its path to avoid obstacles by detecting the position of obstacles using a single sensor. Components of its operations are as follow:

The first component: All feasible waypoints are determined. These points are selected on the condition that they must not be points within the radius of any obstacle and they must follow the path finding criteria: the shortest, smoothest and safest path.

The second component: Feasible waypoints that are not selected are improved to have the desired qualifications. In this study, another set of feasible waypoints is randomly selected and checked whether they met the criteria. If they meet the criteria, they are determined to be waypoints. Otherwise, feasible waypoints are randomly selected again.

The third component: Obstacle avoidance—robots perceive the positions of various obstacles using a single sensor. The sensor detects the coordinates and positions of obstacles and sends the information to robots. When robots realize the coordinates of the obstacles, the paths are improved to prevent robots from colliding with those obstacles.

## Problem statement

**Fig 1:**
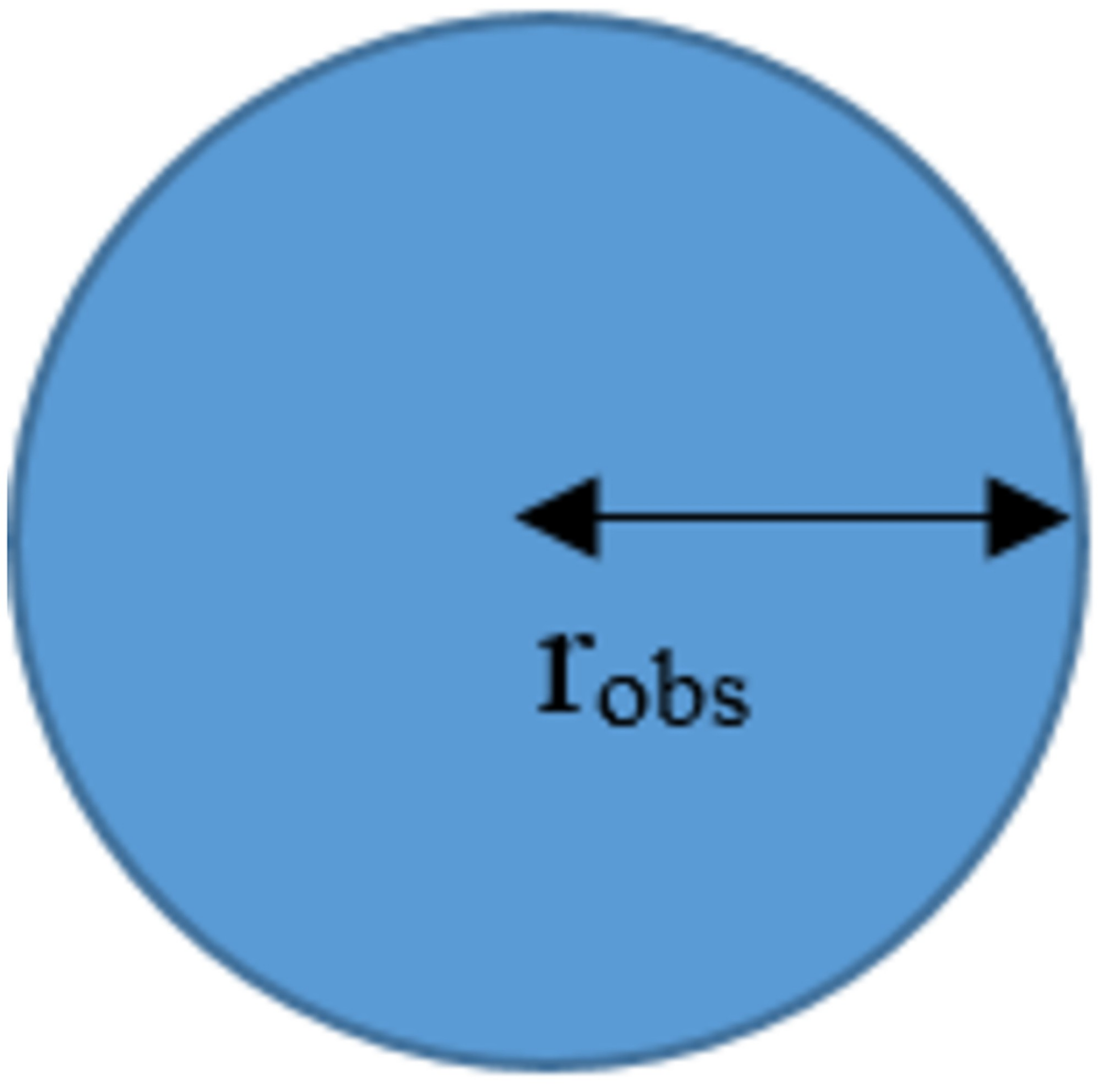
Characteristics of the obstacles.

In this study, a robot was required to move from a starting position to a destination position on a search space consisting of static and moving obstacles on different positions of the search space. The shortest, smoothest and safest path is the path planning target. The requirements for the study are detailed below:

Assumption 1: Obstacles are shown in the form of a circle whose center is the positions on the search space. The radius of the circle (*r*_*obs*_) shows the areas occupied by the obstacles where the robot cannot move into. Examples of obstacles are shown in Fig 1.

**Fig 2:**
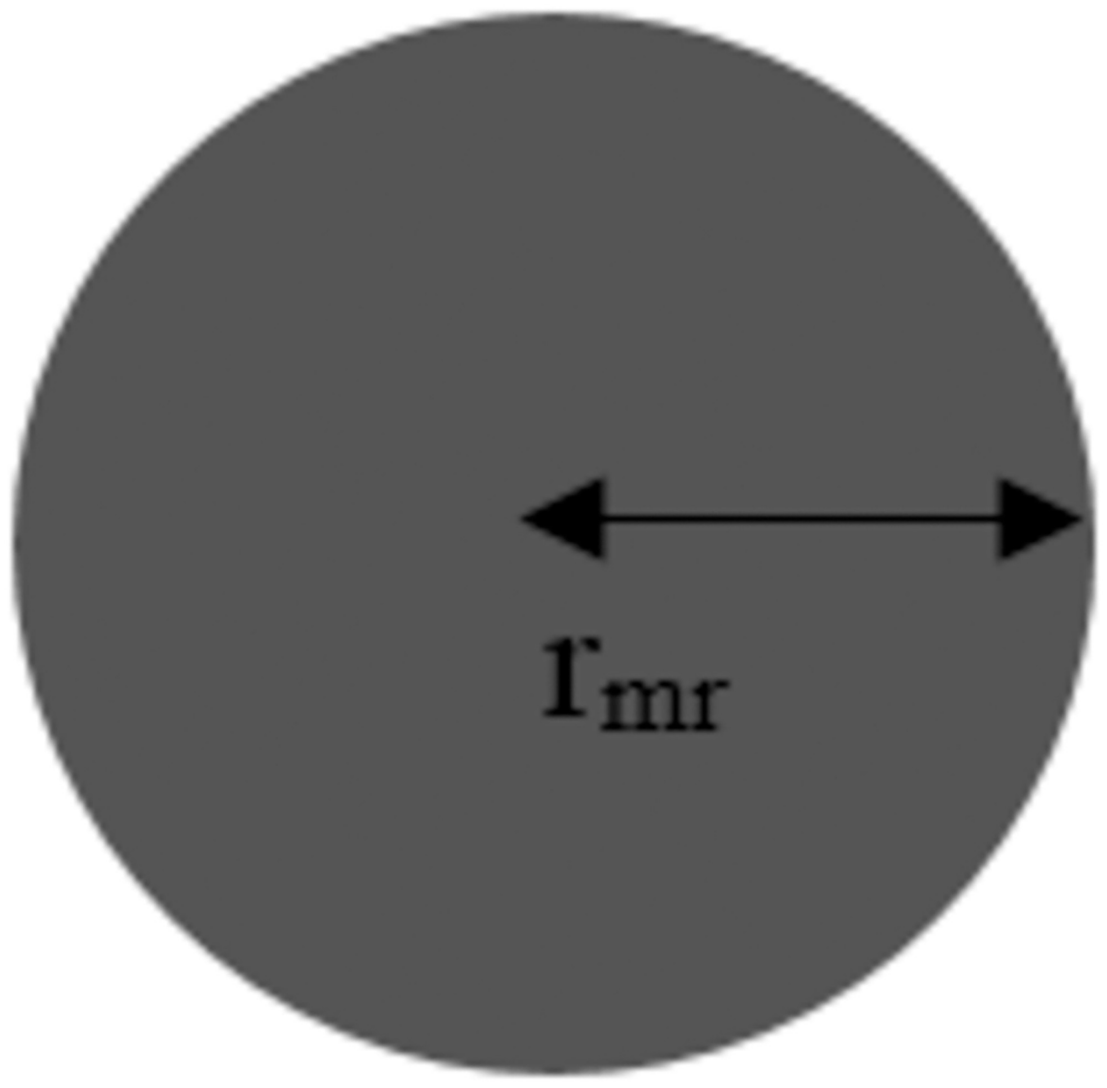
Characteristics of robots.

Assumption 2: The robot is shown in the form of a circle. The size of its radius (*r*_*mr*_) shows the size of the robots, as shown in Fig 2.

**Fig 3:**
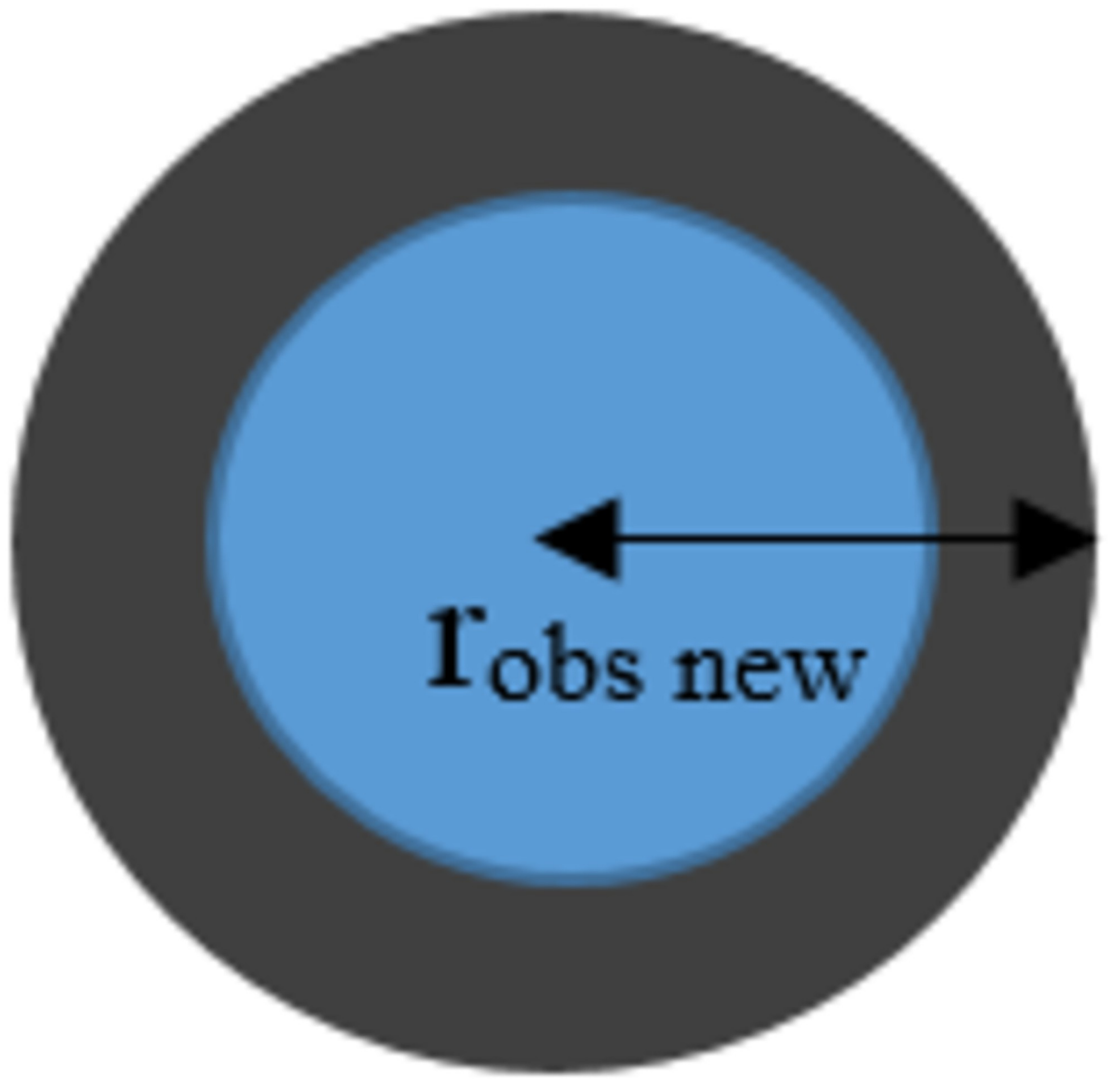
The obstacles when the size of the radius is changed.

In previous studies, robots were often determined as a spot on a search space. Such a determination did not reflect the movement of robots in the real world, and the size of the robots must be taken into consideration. Therefore, in this study, the size of the radius of the robot was combined with the size of the radius of the obstacles to ensure that the movements were consistent with reality. The new size of the radius of the obstacles is obtained from Eq (1). The characteristics of the obstacles when the size of the radius is changed are shown in Fig 3.

$$\begin{array}{c} r_{obsnew}=r_{obs}+r_{mr} \end{array}$$ (1)

where *r*_*obsnew*_ is the new obstacle radius, *r*_*obs*_ is the old obstacle radius, and *r*_*mr*_ is the robot radius.

Fig 3 shows the characteristics of the obstacles having a radius that occupies the area within the blue circle. After the radius of the obstacles is combined with the radius of the robot, the radius of the obstacles is adjusted to be a new radius, which occupies the area inside the blue and gray circles.

Assumption 3: In this study, kinematic constraints are not taken into consideration. Thus, the movement of the obstacles is the only condition that has an effect on the movement of the robot.

**Fig 4:**
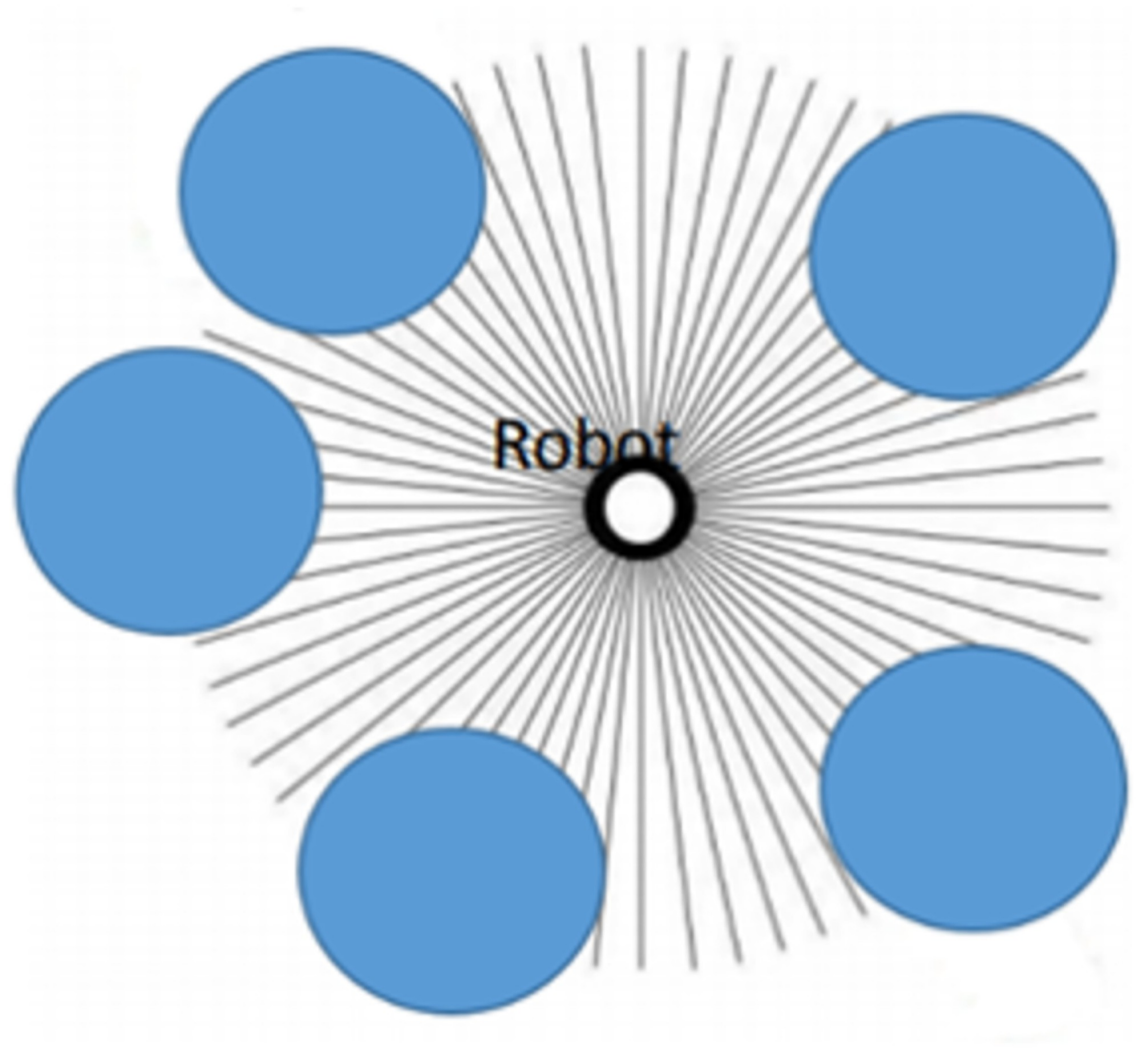
Movement directions of the robot.

Assumption 4: The robot can move all the way around, as seen in Fig 4.

### Criteria for measuring efficiency

#### The shortest path

With regard to path planning, the shortest path is the length of a path from a starting point to a destination point with the minimum value. In each iteration, the algorithm will select a feasible waypoint (*wp*_*j*_(*t*)) to be an answer when the distance between the current waypoint and feasible waypoint has the smallest value. The distance is calculated using the Euclidean algorithm, as shown in Eq (2).

$$\begin{array}{c} d_{t}=\sqrt{{\left(x_{wp_{j}}\left(t+1\right)-x_{wp_{j}}\left(t\right)\right)}^{2}+{\left(y_{wp_{j}}\left(t+1\right)-y_{wp_{j}}\left(t\right)\right)}^{2}} \end{array}$$ (2)

where *d*_*t*_ is the distance between two points, *j* is the ordinal number of feasible waypoints, $x_{wp_{j}}\left(t+1\right)$ is the x coordinate of feasible waypoints in iteration t+1, $x_{wp_{j}}\left(t\right)$ is x coordinate of the current waypoints, $y_{wp_{j}}\left(t+1\right)$ is the y coordinate of feasible waypoints in iteration t+1, and *y*_*wpj*_(*t*) is y coordinate of the current waypoints.

The shortest path calculated from the sum total of the distance between feasible waypoints in each iteration (wpj(2),…,wpj(N-1)) and the current waypoints according to Eq (3).

$$\begin{array}{c} SPL=\Sigma_{t=1}^{N-1}d_{t} \end{array}$$ (3)

where *SPL* is the sum total of the distance between feasible waypoints in each iteration and the starting point and destination point, *d*_*t*_ is the distance between the current waypoints and feasible waypoints in each iteration calculated from Eq (2), and *N* is the number of all waypoints.

**Fig 5:**
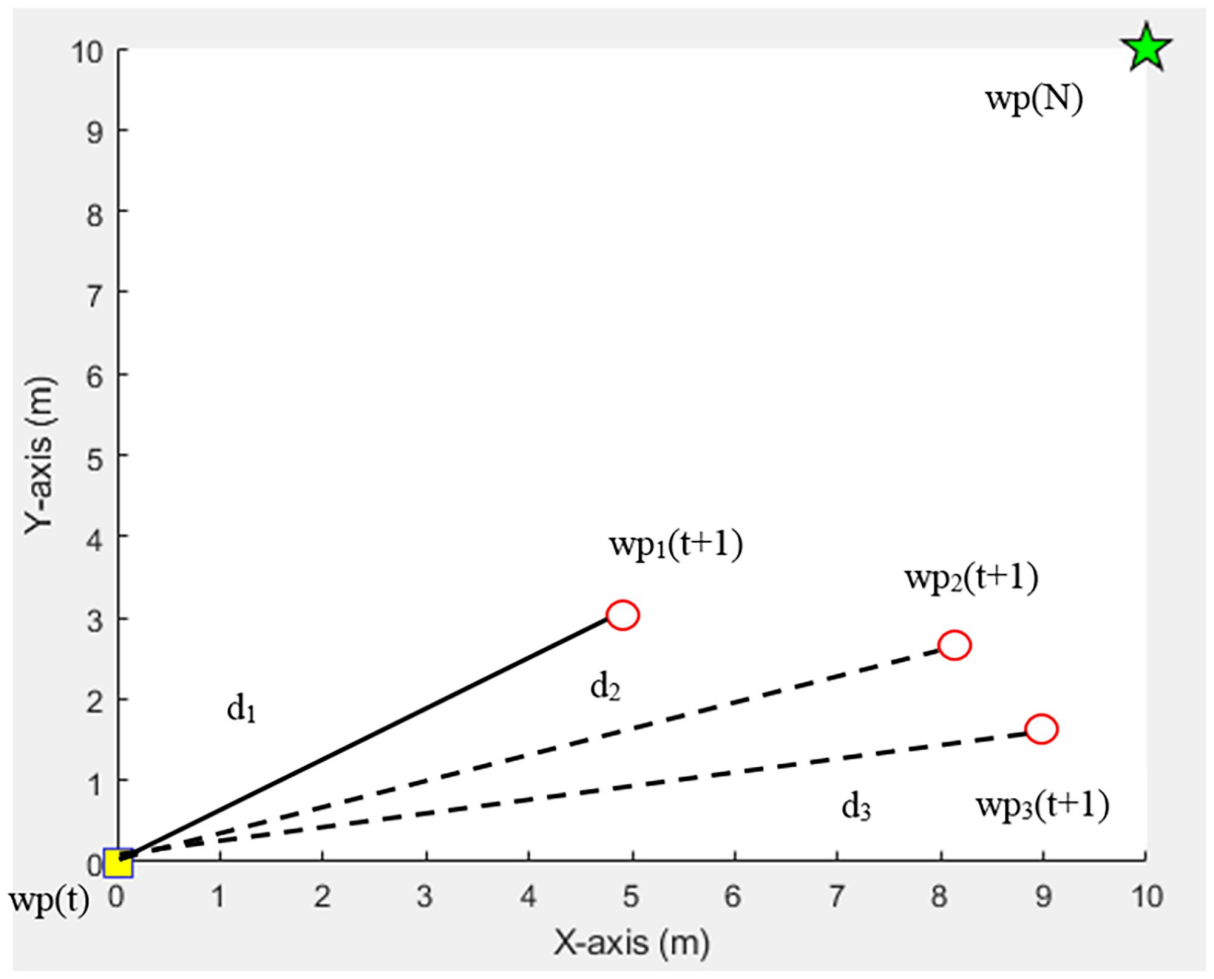
Consideration of the shortest path.

In Fig 5 *d*_1_, *d*_2_, and *d*_3_ are the distances between the current waypoint (*wp*(*t*)) and feasible waypoints *wp*_1_(*t* + 1), *wp*_2_(*t* + 1), and *wp*_3_(*t* + 1), respectively. Point *d*_1_ has the shortest distance. Therefore, the *wp*_1_(*t* + 1) point is considered the next waypoint.

#### Smooth path

**Fig 6:**
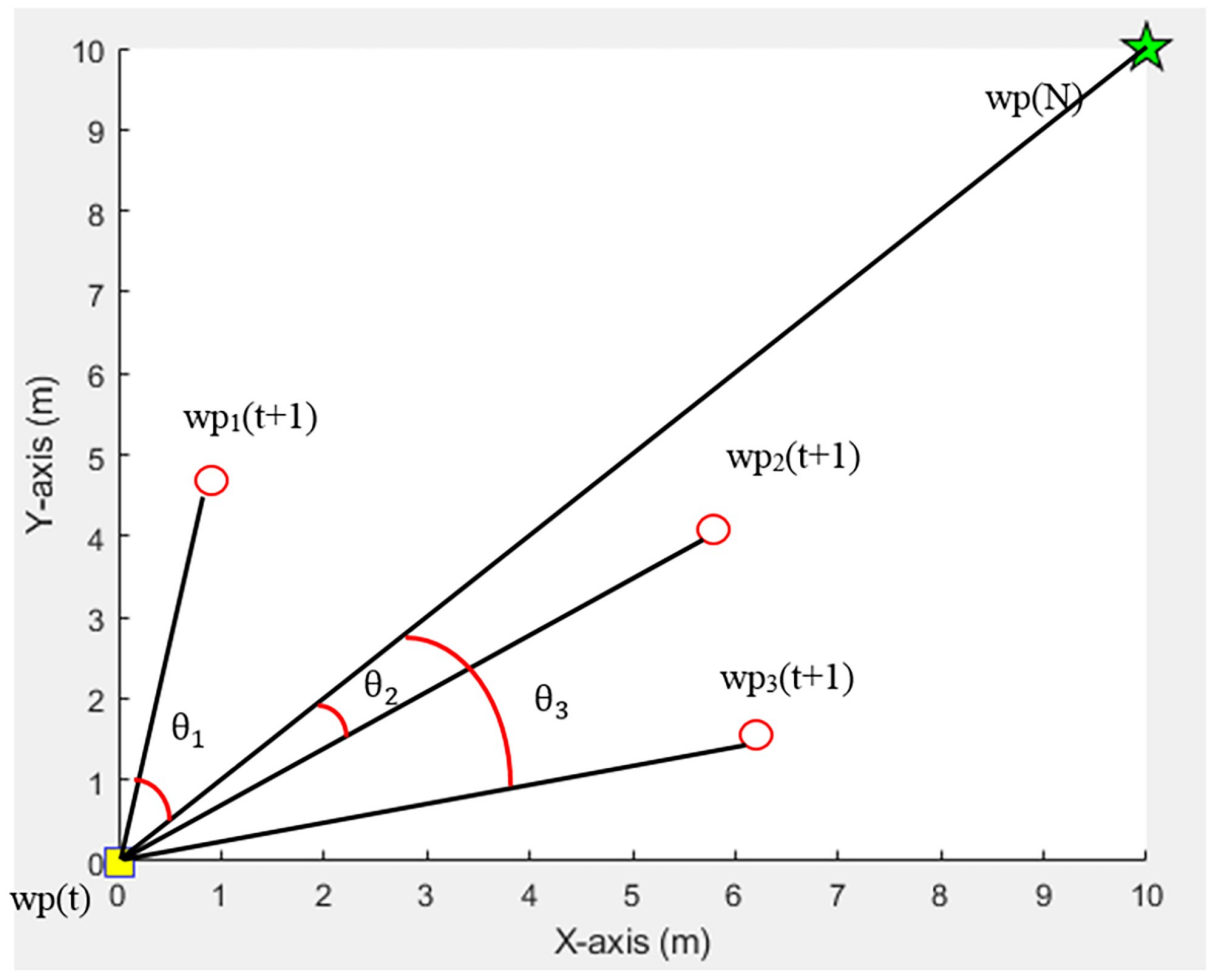
Consideration of smooth paths.

A smooth path is a criterion related to waypoint finding that enables a movement path to be the straightest by calculating the difference between an angle where a straight line connects a current waypoint to a destination and how a part of a straight line that connects between a current waypoint to feasible waypoint react to each other, as seen in Fig 6. The algorithm will pick a waypoint that makes the sum total of the difference between such angles the smallest value according to Eq (4).

$$\begin{array}{c} Smooth=\Sigma_{t=1}^{N-1}\left|\theta_{\left(wp(t),wp(t+1)\right)}-\theta_{\left(wp(t),wp(N)\right)}\right| \end{array}$$ (4)

where *Smooth* is the sum total of the difference of an angle where a feasible waypoint acts on a current waypoint and an angle at a destination acts on a current waypoint, *θ*_(*wp*(*t*), *wp*(*t* + 1))_ is an angle where a feasible waypoint acts on a current waypoint, *θ*_(*wp*(*t*), *wp*(*N*))_ is an angle at a destination acting on a current waypoint, and *N* is all waypoints, $\theta_{\left(wp(t),wp(t+1)\right)}=tan^{-1}\frac{y_{wp_{j}}\left(t+1\right)-y_{wp}\left(t\right)}{x_{wp_{j}}\left(t+1\right)-x_{wp}\left(t\right)}$, and $\theta_{\left(wp(t),wp(N)\right)}=tan^{-1}\frac{y_{wp}\left(N\right)-y_{wp}\left(t\right)}{x_{wp}\left(N\right)-x_{wp}\left(t\right)}$ where *wp*(*t*) is current waypoint, *j* is a sequence of feasible waypoints, *wp*_*j*_(*t* + 1) is a feasible waypoint in iteration *t* + 1, *wp*(*N*) is a destination, $y_{wp_{j}}\left(t+1\right)$ is the y coordinate of the feasible waypoint in iteration *t* + 1, *x*_*wp*_(*t*) is the x coordinate of the current waypoint, $x_{wp_{j}}\left(t+1\right)$ is the x coordinate of the feasible waypoint in iteration *t* + 1, *x*_*wp*_(*t*) is the x coordinate of the current waypoint, *y*_*wp*_(*N*) is the y coordinate of the destination, *x*_*wp*_(*N*) is the x coordinate of the destination, and *N* is the number of total waypoints.

In Fig 6, *θ*_1_, *θ*_2_, and *θ*_3_ are angles at *wp*_1_(*t* + 1), *wp*_2_(*t* + 1), *andwp*_3_(*t* + 1) that act on a destination (*wp*(*N*)). It can be seen that *wp*_2_(*t* + 1) has a smaller angle to the destination than the other points (*wp*_1_, *wp*_3_). Consequently, this point is considered the next waypoint.

#### Safe path

A safe path refers to a movement path that shall have the farthest distance from each obstacle in each work iteration. The algorithm will pick feasible waypoints that give the maximum distance value from each obstacle. A sum total of the distance between paths and each obstacle can be calculated according to Eq (5).

$$\begin{array}{c} Safety=\Sigma_{i=1}^{N-1}\Sigma_{j=1}^{m}\left|wp_{i},O_{j}\right| \end{array}$$ (5)

where *Safety* is the sum total of the distance between feasible waypoints and obstacles, *wp*_*i*_ is a feasible waypoint, *O*_*j*_ is the position of each obstacle center, |*wp*_*i*_, *O*_*j*_| is the distance between feasible waypoints and obstacles, *N* is the number of waypoints, *m* is the number of all obstacles, *i* is a sequence of feasible waypoints, and *j* is a sequence of obstacles.

**Fig 7:**
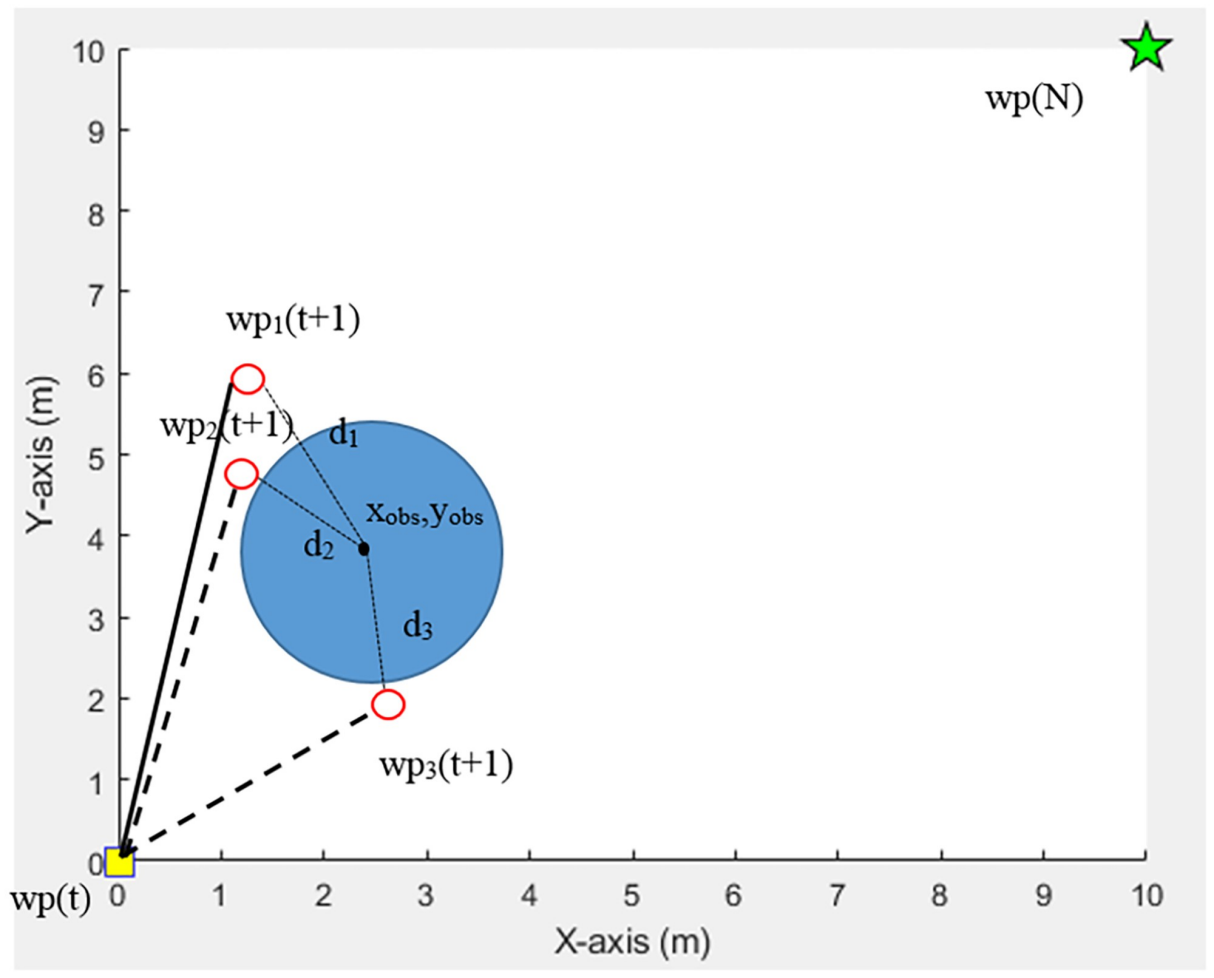
Consideration of safe paths.

In Fig 7, *d*_1_, *d*_2_, and *d*_3_ are the distances between the center point of the obstacle(*x*_*obs*_, *y*_*obs*_) and feasible waypoints *wp*_1_(*t* + 1), *wp*_2_(*t* + 1), and *wp*_3_(*t* + 1), respectively. Point *d*_1_ has the maximum distance. As a consequence, point *wp*_1_(*t* + 1) is considered the next waypoint.

Generally, multi-objective optimization problem solving is divided into two approaches: as an ideal multi-objective optimization procedure, which collects the most suitable answers to each objective for consideration to pick the only best answer again, and as a preference-based multi-objective optimization procedure. All objective functions are reduced to be only one function. After that, a suitable answer is searched for accordingly [37].

In this article, the proposed algorithm must select only one suitable waypoint. Thus, the preference-based multi-objective optimization procedure is used to find the best fit paths. The following 3 criterias are used to find the best fit paths by determining the fitness function according to the following equation:

$$\begin{array}{c} Fitness=\left(w_{1}*SPL\right)+\left(w_{2}*Smooth\right)+\left(w_{3}*Safety\right) \end{array}$$ (6)

where *Fitness* is the fitness value calculated from the fitness function, is the sum total of the distance between feasible waypoints in each iteration and a starting point and a destination point, as calculated from Eq (3), *Smooth* is the sum total of the difference of an angle where feasible waypoints act on current waypoints and an angle where destinations act on current waypoints, as calculated from Eq (4), *Safety* is the sum total of the distance between feasible waypoints and obstacles, as calculated from Eq (5), *w*_1_, *w*_2_, and *w*_3_ are the weighted values of *SPL*, *Smooth* and *Safety*, respectively. In addition, the path given by the proposed algorithm is a path that does not cause collisions with obstacles as it takes into account only feasible points of motion to create an optimal path. These feasible points are then calculated using the fitness function according to Eq (6). The proposed algorithm divides the possible points of motion that are not considered as points of motion into two cases. First, a feasible waypoint is positioned within the radius of the obstacle. Such displacement points are not taken to calculate fitness. meanwhile A new feasible waypoint will be randomly selected in another set. If the criteria are met, these points will be designated as the next waypoint. Another case occurs when an obstacle blocks a feasible waypoint. As a result, such displacement points will not be included in the fitness calculation either. The algorithm randomly selects one set of feasible waypoints. In the same way as in the first case. Both methods are explained in more detail in the section of handling of infeasible waypoints.

Weight determination for each objective depends on the importance given to that objective. In this study, primary importance was given to path length since it was found in the literature review that studies about path planning were most likely related to finding the shortest path since this helps reduce fuel costs and travel time for the robots. Thus, the *w*_1_ weight value is determined to have the maximum value, and *w*_2_ = *w*_3_. However, the sum total of all weight values must be equal to 1 according to the following equation:

$$\begin{array}{c} w_{1}+w_{2}+w_{3}=1 \end{array}$$ (7)

where *w*_1_, *w*_2_, and *w*_3_ are weighted values of SPL, Smooth and Safety, respectively.

Fitness values calculated from the fitness function can be varied to serve convenience in comparing efficiency from fitness values. Therefore, the fitness value should be adjusted to be no larger than 1 by recalculating the fitness value from Eq (7) according to the following equation:

$$\begin{array}{c} Fitness_{new}=\frac{1}{Fitness} \end{array}$$ (8)

where *Fitness*_*new*_ is the recalculated fitness value, and *Fitness* Fitness is the old fitness value.

### Movement of obstacles

In this study, obstacles are determined to be in various positions as required. With regard to path planning in a dynamic environment, each obstacle is determined to move with 2 models: linear movement and nonlinear movement. Each movement model is detailed as follows.

#### Linear movement obstacles

In linear movement, obstacles move along a straight line according to the required velocity and directions, as shown in the following equations:

$$\begin{array}{c} x_{obsNew}=x_{obs}+v_{obs}\times COS\varphi_{obs} \end{array}$$ (9)

$$\begin{array}{c} y_{obsNew}=y_{obs}+v_{obs}\times Sin\varphi_{obs} \end{array}$$ (10)

where *x*_*obsNew*_ is the new obstacle position on the x-axis, *y*_*obsNew*_ is the new obstacle position on the y-axis, *x*_*obs*_ is the old obstacle position on the x-axis, *y*_*obs*_ is the old obstacle position on the y-axis, *v*_*obs*_ is the velocity of the obstacle, and *φ*_*obs*_ is the direction of the obstacle.

#### Nonlinear movement obstacles

**Fig 8:**
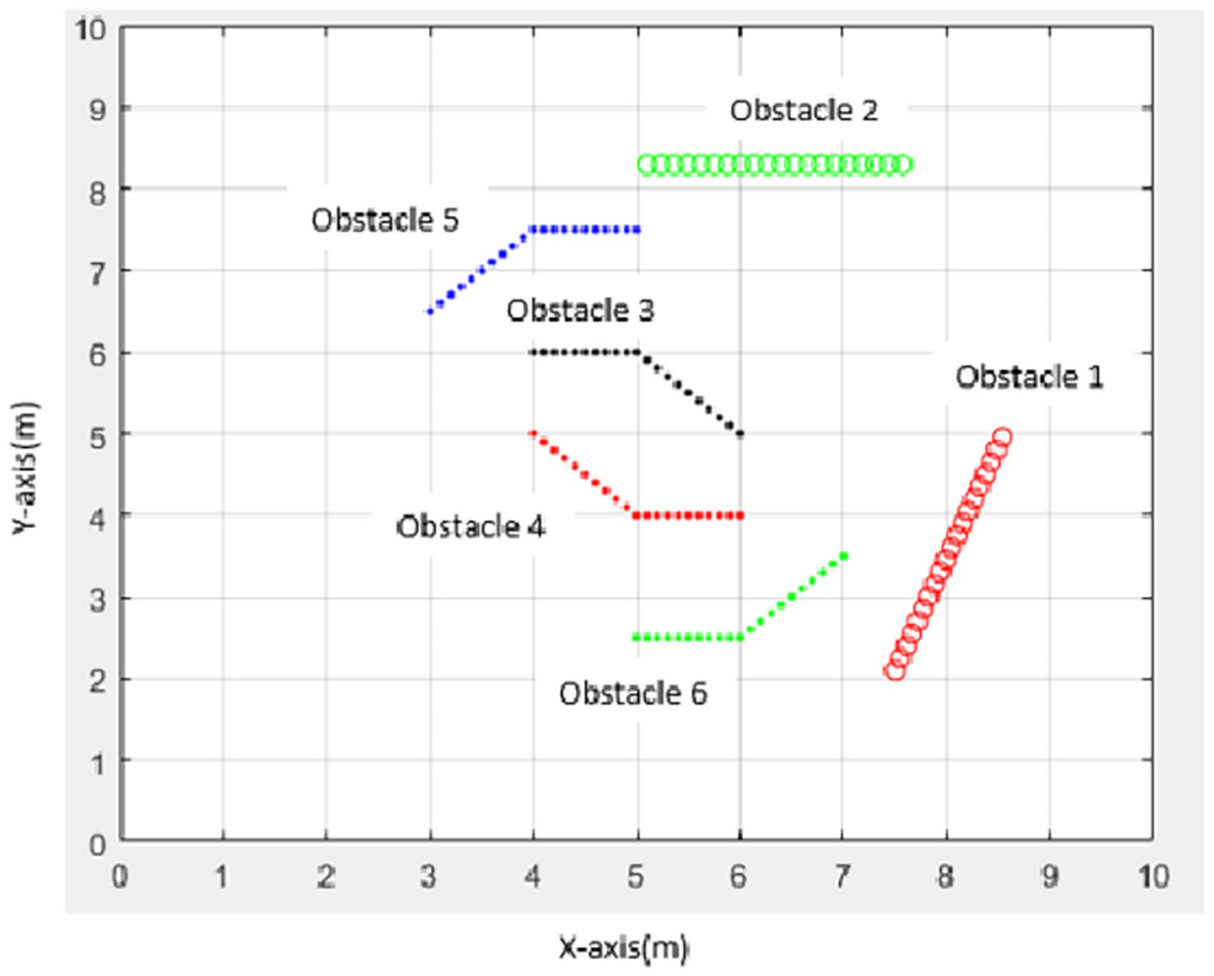
The movement of each obstacle in a dynamic environment.

In nonlinear movement, obstacle movement is divided into two phases: obstacles move along a straight line and obstacles move along a diagonal line to simulate obstacle movements closer to a real situation. The movement directions of each obstacle in the dynamic environment are shown in Fig 8.

## Proposed algorithm

### MOEPSO path planning

In this section, multi-objective evolutionary particle swarm optimization (MOEPSO) is developed for robot path planning. As mentioned earlier, robot path planning is an important process for robot navigation. In addition, it is a popular topic for study in the robotics field.

**Fig 9:**
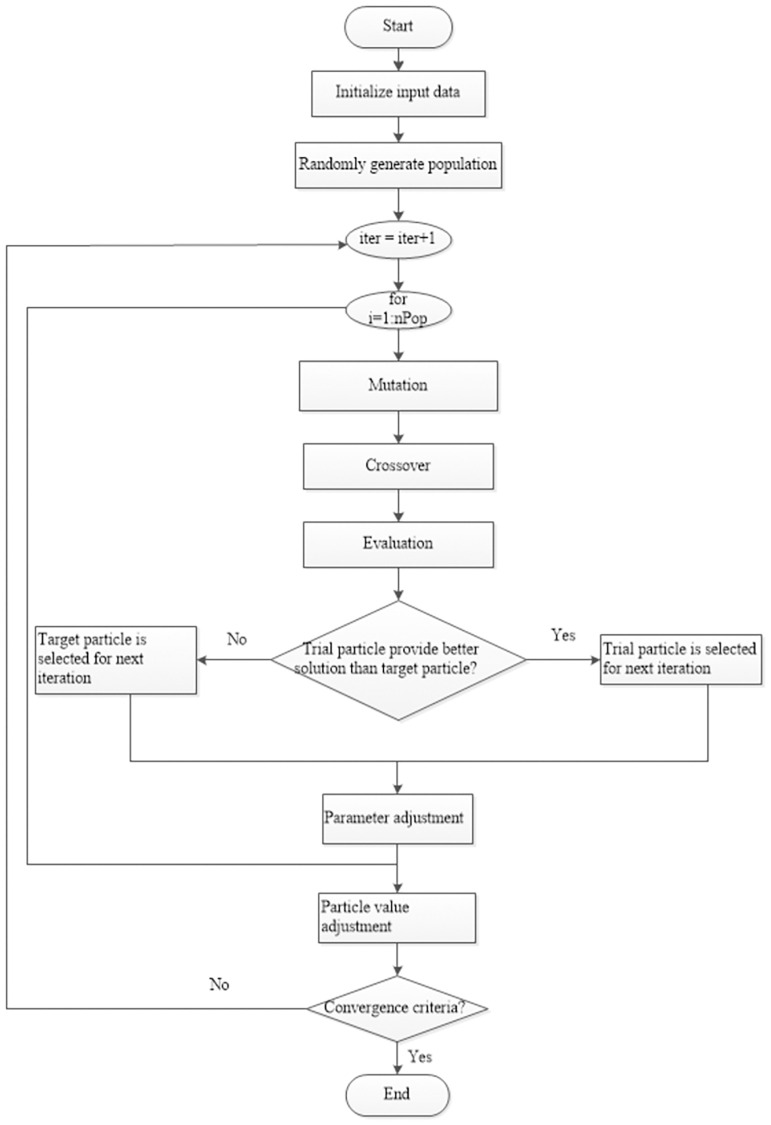
The MOEPSO flowchart.

For the path planning objective in this study, the robot must achieve the shortest, smoothest and safest path in both static environments and dynamic environments. The robot must perceive positions and be able to avoid existing obstacles. The MOEPSO algorithm is applied to solve this problem. First, path planning problems are transformed into MOEPSO-based problems by the requirements of various objectives, as described in the problem statement. In each iteration of the algorithm, feasible waypoints are created. They are selected in accordance with objectives determined for building paths to destinations. When the robot does not encounter obstacles, it moves along the paths to the destinations. The operating procedures of the algorithm are shown in Fig 9.

**Fig 10:**
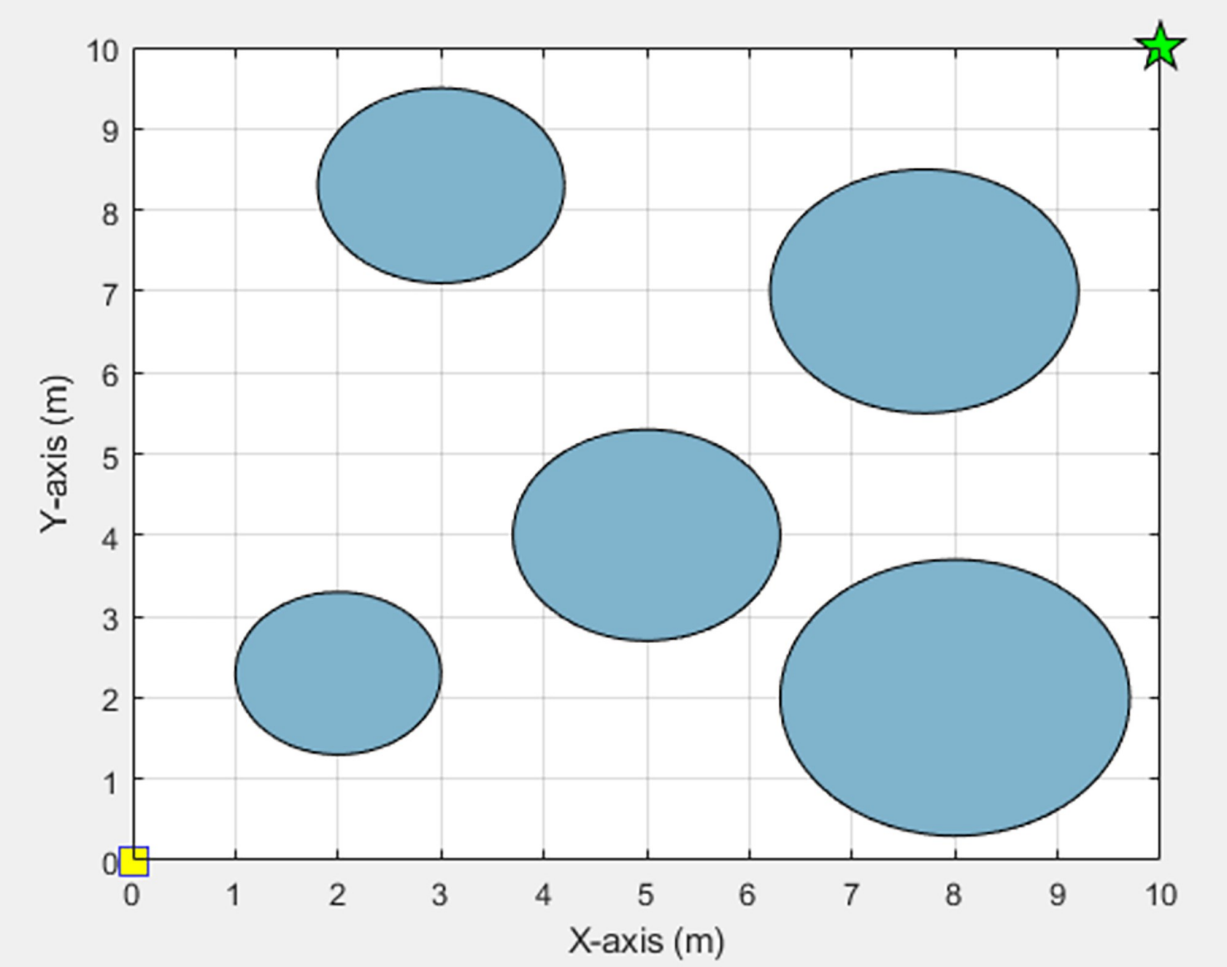
Example of the traveling environment.

Procedure 1 Initialization: Determine a starting point and destination point of the movement. An environment associated with the movement is created that consists of a starting point, destination, and obstacles. For the convenience of the experiment, the traveling environment is simulated in the form of an n × n table [12]. Obstacles are represented with blue circles. The starting point is represented with a yellow square, and the destination is represented with a green star. Their positions are determined on the table by the requirements of each experiment. An example of the traveling environment is shown in Fig 10.

Procedure 2—Particle generation: Randomly generate initial population. In this procedure, nPop initial particles are created. They are the determined position values and velocity with randomization. Various values are determined for each particle as follows: $X_{j}^{it}$ is the position of particle j in iteration it, $V_{j}^{it}$ is the velocity of particle j in iteration it, $Pbest_{j}^{it}$ is the best position of particle j in iteration it, and *F*(*X*_*j*_) is the fitness value of the particle where *j* = 1, 2, …, *nPop* is a sequence of particles, *it* = 1, 2, …, *Maxit* is the number of iterations of the algorithm.

Procedure 3—Mutation: After each particle is created, Mutant Particle *M*_*j*_ will be created by the following equation:

$$\begin{array}{c} M_{j}^{it}=X_{r_{1}^{i}}^{it}+F*\left(X_{r_{2}^{i}}^{it}-X_{r_{3}^{i}}^{it}\right) \end{array}$$ (11)

where $M_{j}^{it}$ is the mutant particle in iteration it, $r_{1}^{i},r_{2}^{i},$ and $r_{3}^{i}$ is a randomly selected integer in the range of [1,nPop], *F* is a positive integer randomly selected from [*F*^*Min*^, *F*^*Max*^], and *X* is an old particle by the time the initial population is created.

Procedure 4—Crossover: After the particles are mutated, crossover operators will be used to make trial particle T from each particle and its mutant particle. The trial particle will be created by the following equation:

$$\begin{array}{c} T_{j}^{it}\{\begin{array}{cc} M_{j}^{b} & if\left(rand_{b}\left[0,1\right)\leq CR\right)or\left(b=b_{rand}\right)\\ X_{j}^{b} & otherwise\\ b=1,2,\cdots ,DM \end{array} \end{array}$$ (12)

where $T_{j}^{it}$ is a trial particle j in iteration it, $M_{j}^{b}$ is a mutant particle j in dimension b, *CR* is the crossover ratio, which must be in the range of [0, 1], *b*_*rand*_ is a randomly selected integer in the range of [1,DM], *DM* is the number of decision variable dimensions, and *j* is a particle sequence.

Procedure 5—Evaluation of fitness values of particles and selection: Each particle, including the existing trial particles, will be evaluated for fitness values calculated from the objective function according to Eq (8). Particles with better fitness values will be selected to be populations for the next iteration.

Procedure 6—Particle value adjustment: In this procedure, each particle will be adjusted to the personal best value, global test value, and position value according to Eqs (13) and (14)

$$\begin{array}{c} X_{j}^{it+1}=X_{j}^{it}+V_{j}^{it+1} \end{array}$$ (13)

where $X_{j}^{it+1}$ is the new position of the particle, $X_{j}^{it}$ is the old position of the particle, and $V_{j}^{it+1}$ is the new velocity of the particle.

$$\begin{array}{c} V_{j}^{it+1}=w*V_{j}^{it}+C_{1}Rand_{1}\left(\right)*\left(Pbest_{j}^{it}-X_{j}^{it}\right)+C_{2}*Rand_{2}\left(\right)*\left(Gbest-X_{j}^{it}\right) \end{array}$$ (14)

where $V_{j}^{it+1}$ is the new velocity of the particle, *w* is the weighted value of the movement *C*_1_ and *C*_2_ are weighted values for particle-based learning and weighted values for group-based learning, respectively, *Rand*_1_() and *Rand*_2_() are real numbers randomly selected in the range of [0, 1], $Pbest_{j}^{it}$ is the best position of each particle, $X_{j}^{it}$ is the old position of the particle, and Gbest is the best position of the swarm.

Procedure 7—Parameter adjustment: Three parameters whose values have an effect on the working efficiency of the EPSO algorithm are the weighted value of movement (*w*), the weighted value of particle-based learning (*C*_1_) and the weighted value of group-based learning (*C*_2_). All three parameters will be adjusted according to Eqs (15)–(17)

$$\begin{array}{c} w^{it}=0.5*\left(w^{Max}+w^{Min}\right)+K_{w}*[arctan(\frac{2\pi }{MaxIt}*it+\pi )]*(w^{Max}-w^{Min}) \end{array}$$ (15)

$$\begin{array}{c} C_{1}^{it}=0.5*\left(C_{1}^{Max}+C_{1}^{Min}\right)+K_{C_{1}}*[arctan(\frac{2\pi }{MaxIt}*it+\pi )]*(C_{1}^{Max}-C_{1}^{Min}) \end{array}$$ (16)

$$\begin{array}{c} C_{2}^{it}=0.5*\left(C_{2}^{Max}+C_{2}^{Min}\right)+K_{C_{2}}*[arctan(\frac{2\pi }{MaxIt}*it+\pi )]*(C_{2}^{Max}-C_{2}^{Min}) \end{array}$$ (17)

where *w*^*it*^ is the weighted value of movement in iteration it, *w*^*Max*^ is the maximum weighted value, *w*^*Min*^ is the minimum weighted value, *MaxIt* is the whole number of iterations, $C_{1}^{it}$ is the weighted value for particle-based learning in iteration it, $C_{1}^{Max},$ is the maximum weighted value of particle-based learning, $C_{1}^{Min}$ is the minimum weighted value of particle-based learning, $C_{2}^{it}$ is the weighted value for group-based learning in iteration it, $C_{2}^{Max}$ is the maximum weighted value for group-based learning, $C_{2}^{Min}$ is the minimum weighted value for group-based learning, *K*_*w*_ is the factor value of the weighted value of movement, $K_{C_{1}}$ is the factor value of the weighted value for particle-based learning, and $K_{C_{2}}$ is the factor value of the weighted value for group-based learning.

Parameter adjustment using the conventional process causes the distance from a starting point to a destination point to have a high value, which is contrary to the objective of path planning, which is expected to obtain the shortest path. Consequently, the process of parameter adjustment is improved by adjusting the weighted value of movement in each iteration according to the following equation:

$$\begin{array}{c} w^{it+1}=w^{it}*N(0,1) \end{array}$$ (18)

where *w*^*it*+1^ is the w value in iteration it + 1, *w*^*it*^ is the w value in iteration it, *N*(0, 1) is the real number randomly selected from the normal distribution in which the mean is equal to 0 and the variance is equal to 1, and *it* is 1,2,⋯, *MaxIt*.

**Fig 11:**
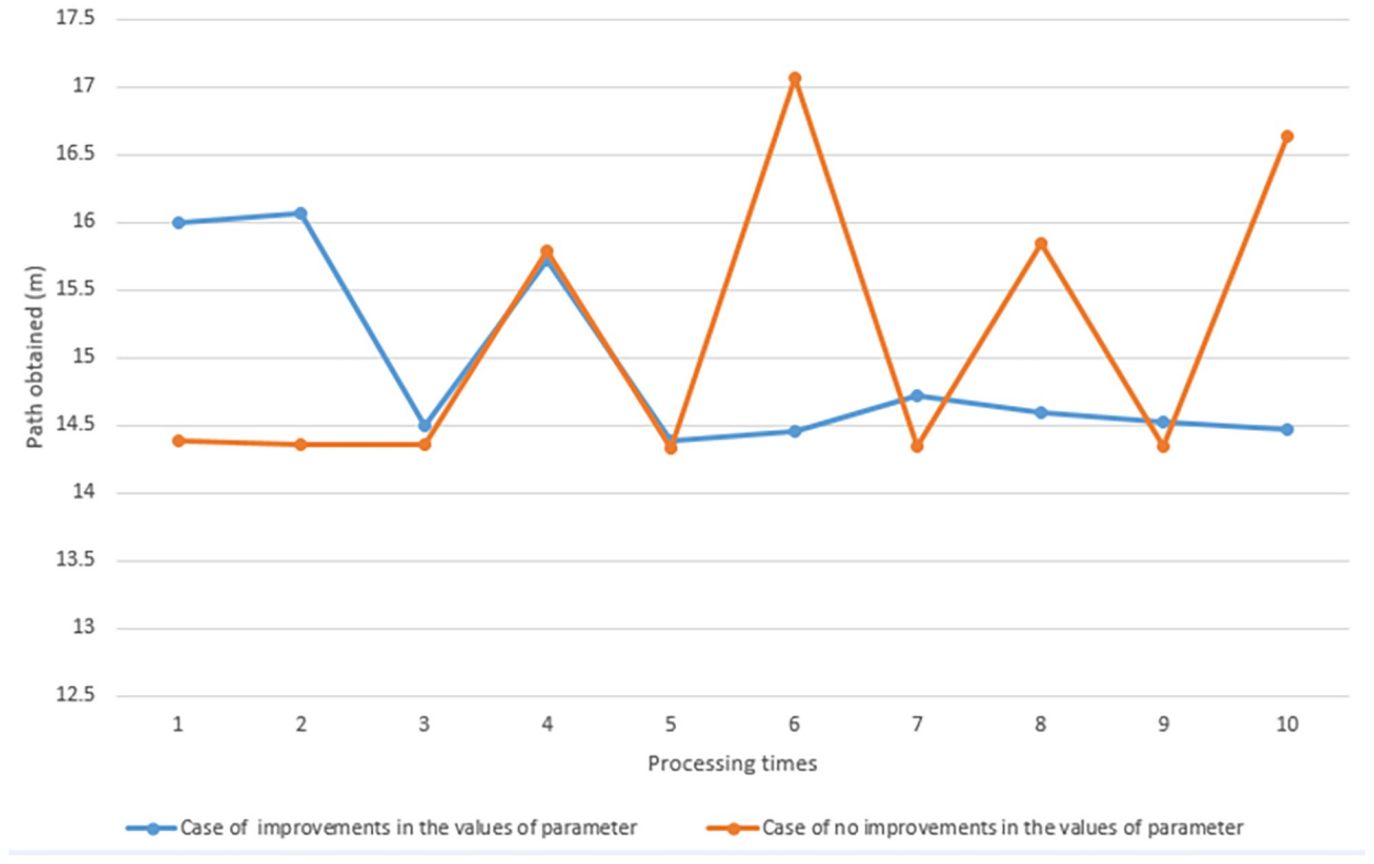
Comparison of path obtained from the proposed algorithm.

In this work, all three parameters whose values have an effect on the working efficiency of the EPSO algorithm are validated by the proposed algorithm. The result shows that parameter changing in each iteration makes a better path as shown in the Fig 11.

From the Fig 11, it is a graph showing the comparison of the distance obtained by the proposed algorithm. The algorithm was processed in a static environment with 4 obstacles, the algorithm was processed 10 times. The orange curve shows the distance obtained by the algorithm in the absence of improvements for the values of *w*, *C*_1_, and *C*_2_ each iteration. The blue curve shows the distance obtained by the algorithm in case of improvements in the values of *w*, *C*_1_, and *C*_2_ each iteration. The graph shows that The distance obtained by the algorithm with adjusted w, C1 and C2 values at each cycle is a better result. In addition, the distance obtained tends to get a smaller value with the number of executions. Whereas the distances obtained by the algorithm without improvements for *w*, *C*_1_, and *C*_2_ are given longer distances and It tends to get more distances based on the number of processes. In conclusion, the parameter changing in each iteration result to a better path. In the PSO algorithm, the three coefficient parameters are *w*, *C*_1_, and *C*_2_ that are held at constant values during the search process for all iterations. However, dynamic adjustment of these coefficient parameters according to the number of iteration is expected to achieve better results. At the beginning of search, a large *w* contributes to more effective exploration at global level, and at the later iterations, the exploitation at local search can be greatly enhanced by small inertia weight. The cognitive weight *C*_1_ signifies the affection of personal best experience. At the beginning of search, this coefficient should be large to enhance the exploration. But, as the final iterations are approached, it should be smaller to improve exploitation. This process implies that at the beginning of search, a particle position relies more on the past individual experience instead of the best position of the whole swarm, while the opposite occurs towards the end. The social weight *C*_2_ applies the affection of best position of the whole swarm. To prevent a particle from getting trapped in a local optimal point, the influence of this parameter should be enhanced according to the increasing iterations. At beginning, this parameter should have little influence on the particle position. While at later iterations, this parameter should be large in order to enhance the social communication between the particle and swarm. All three parameters will be adjusted according to Eqs (15)–(17).

### Handling of infeasible waypoints

In each iteration of the algorithm, feasible waypoints are created to build paths from a starting point to a destination. These feasible waypoints will be evaluated for fitness values. If they are determined to be infeasible waypoints, they will not be used to build paths for the robot. The conditions used to determine infeasible waypoints are detailed below.

#### Waypoints in obstacles

**Fig 12:**
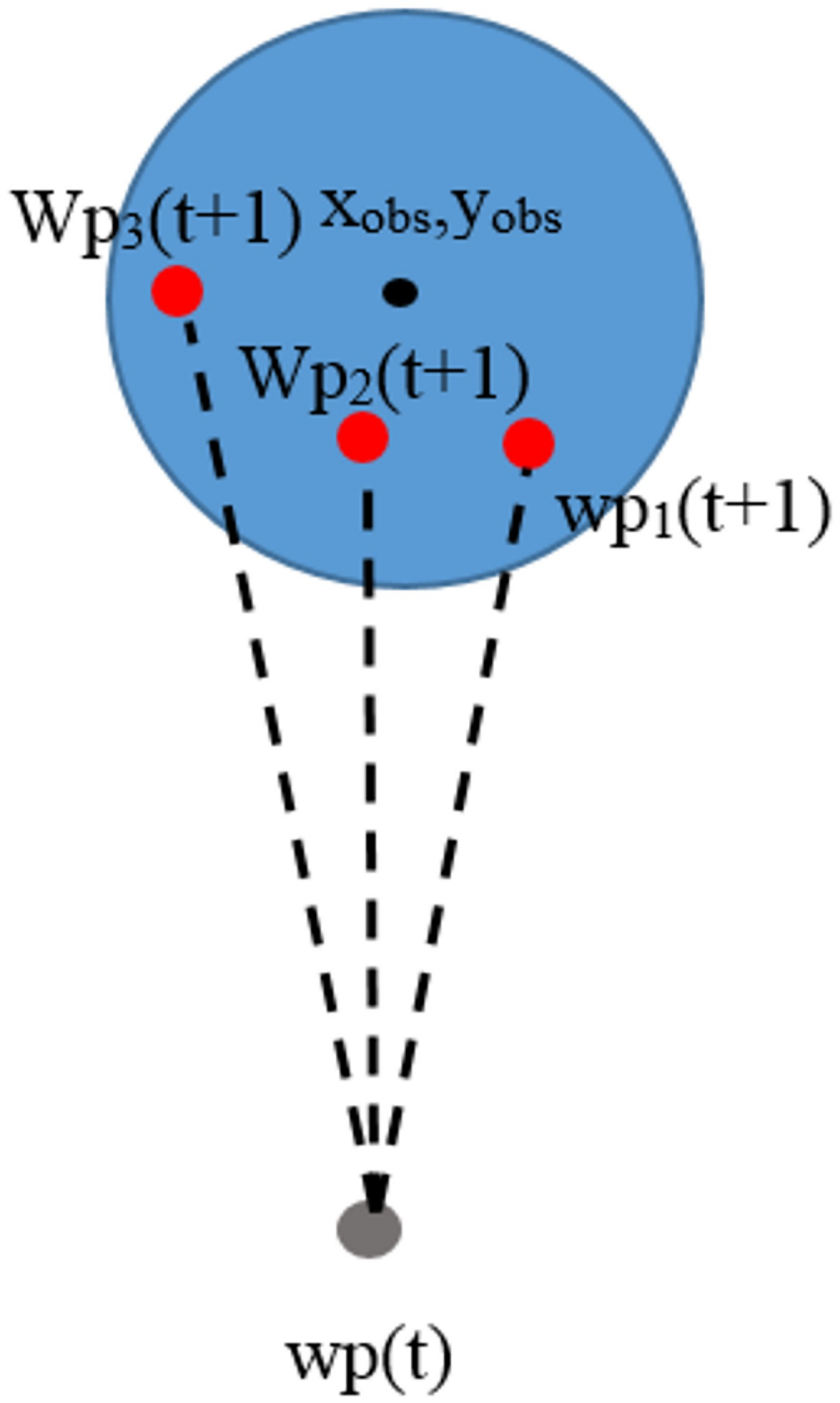
Infeasible waypoints when the points are in obstacles.

Events in which feasible waypoints are in obstacles are shown in Fig 12. They are tested by calculating the distance between feasible waypoints and the center of obstacles using the Euclidean distance method. If the obtained distance is less than the radius of the obstacles, those points will be determined as infeasible waypoints and will not be used to build paths.

**Fig 13:**
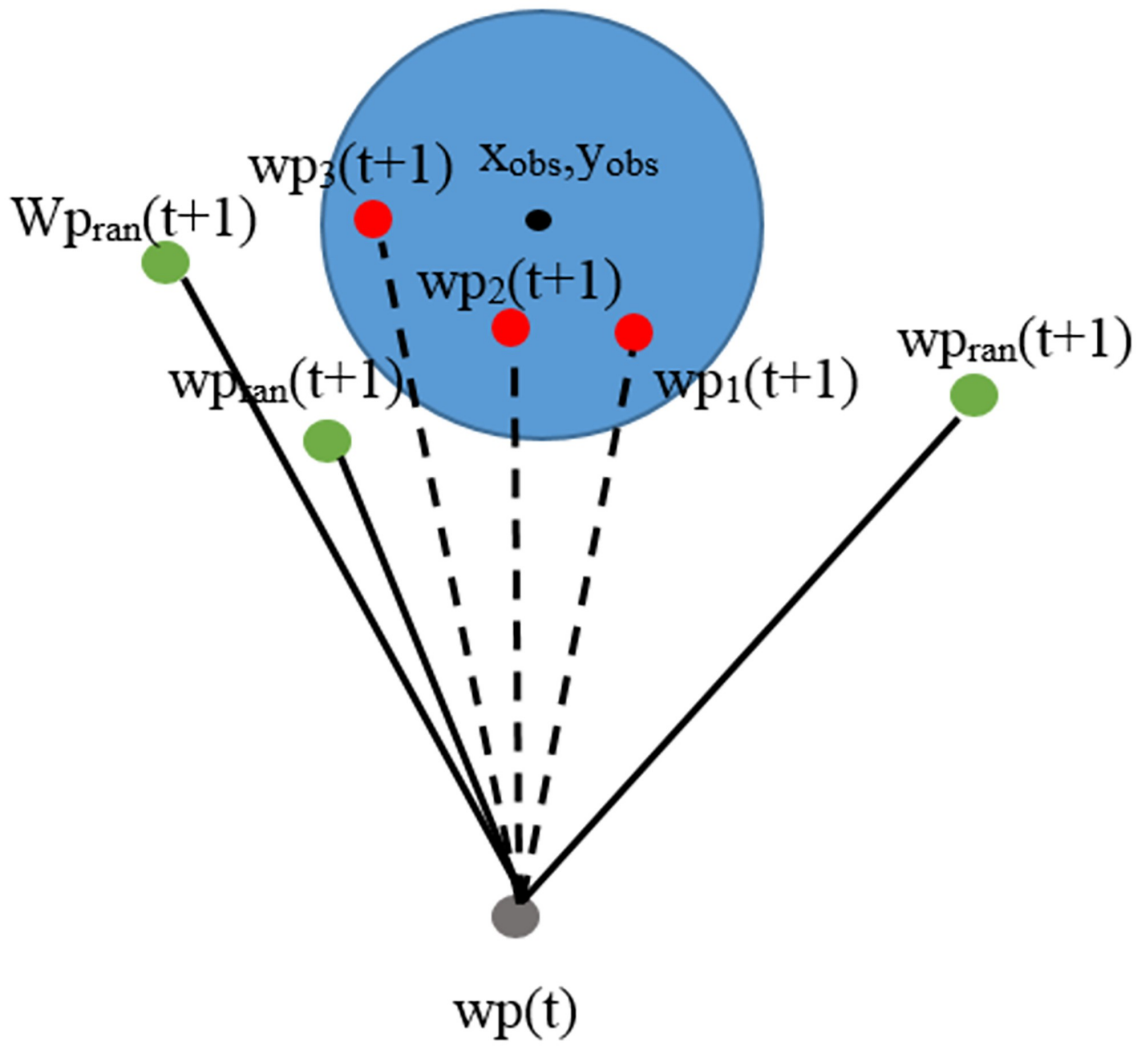
Infeasible waypoint handling when points are in obstacles.

In Fig 12, the blue circles represent obstacles in the environment. The center of the obstacles is determined to be in the position (*X*_*obs*_, *Y*_*obs*_). Current waypoints (*wp*(*t*)) are represented with gray spots. Feasible waypoints are represented with 3 red spots as*wp*_1_*t* + 1, *wp*_2_(*t* + 1), and *wp*_3_(*t* + 1). They will be calculated to find the distance between the center of the obstacles and the positions of each point. If the distance of any point is less than the radius of the obstacles, it will be determined to be an infeasible waypoint, making it impossible to build a path. In the figure, it can be seen that all 3 feasible waypoints have distances less than the radius of the obstacles. Therefore, the algorithm is unable to connect paths from the current waypoint to those three points. Infeasible waypoint handling is described in Fig 13.

In Fig 13, it is noticeable that the feasible waypoints *wp*_1_(*t* + 1), *wp*_2_(*t* + 1), and *wp*_3_(*t* + 1) have distances less than the radius of the obstacles, making them infeasible waypoints, and paths are unable to be connected from the current waypoint (wp(t) to their positions. In this study, the problem handling method is proposed by randomly selecting new feasible waypoints, namely, *wp*_*ran*_(*t* + 1) represented with green spots. The randomly selected points will be calculated to find the distance between the center of the obstacles and their positions. If the distance is greater than the obstacle radius, they will be determined to be waypoints, and paths can be connected from the current waypoint to them. Otherwise, feasible waypoints shall be randomly selected again.

#### Obstacle block paths

**Fig 14:**
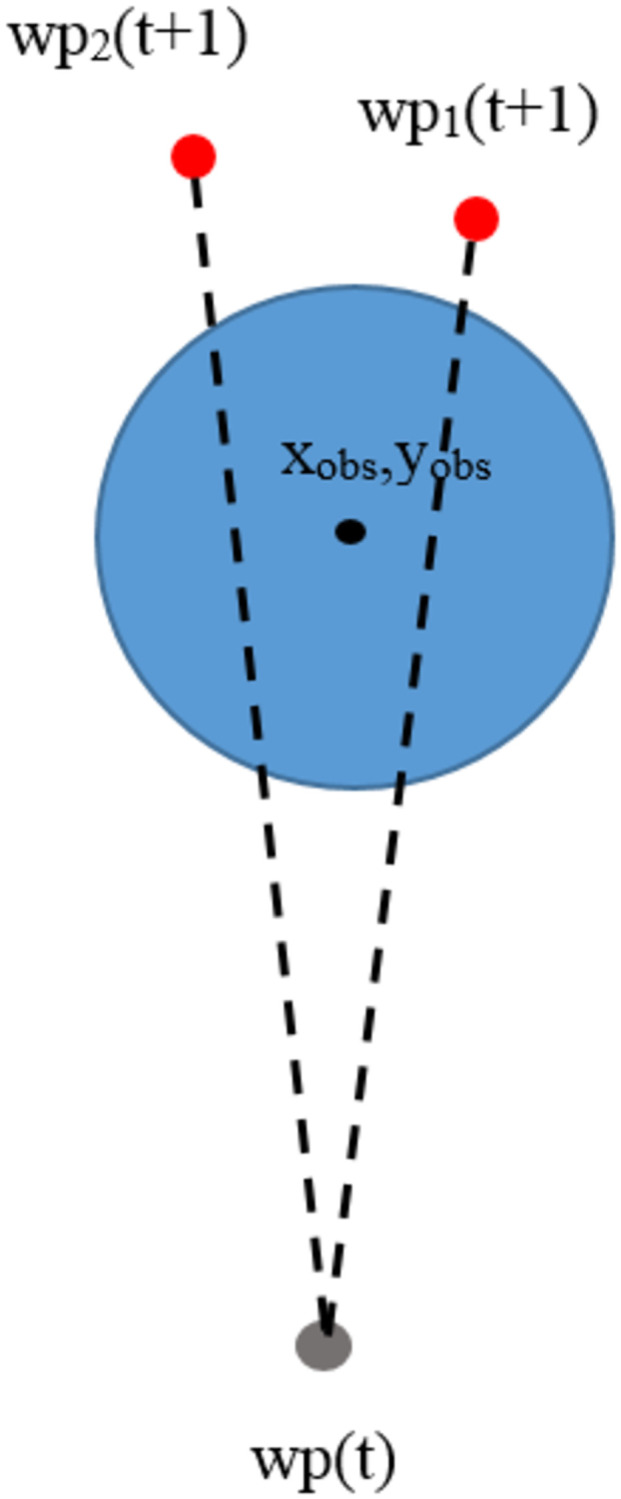
Infeasible waypoints when obstacles block paths.

Events with obstacle block paths are shown in Fig 14.

**Fig 15:**
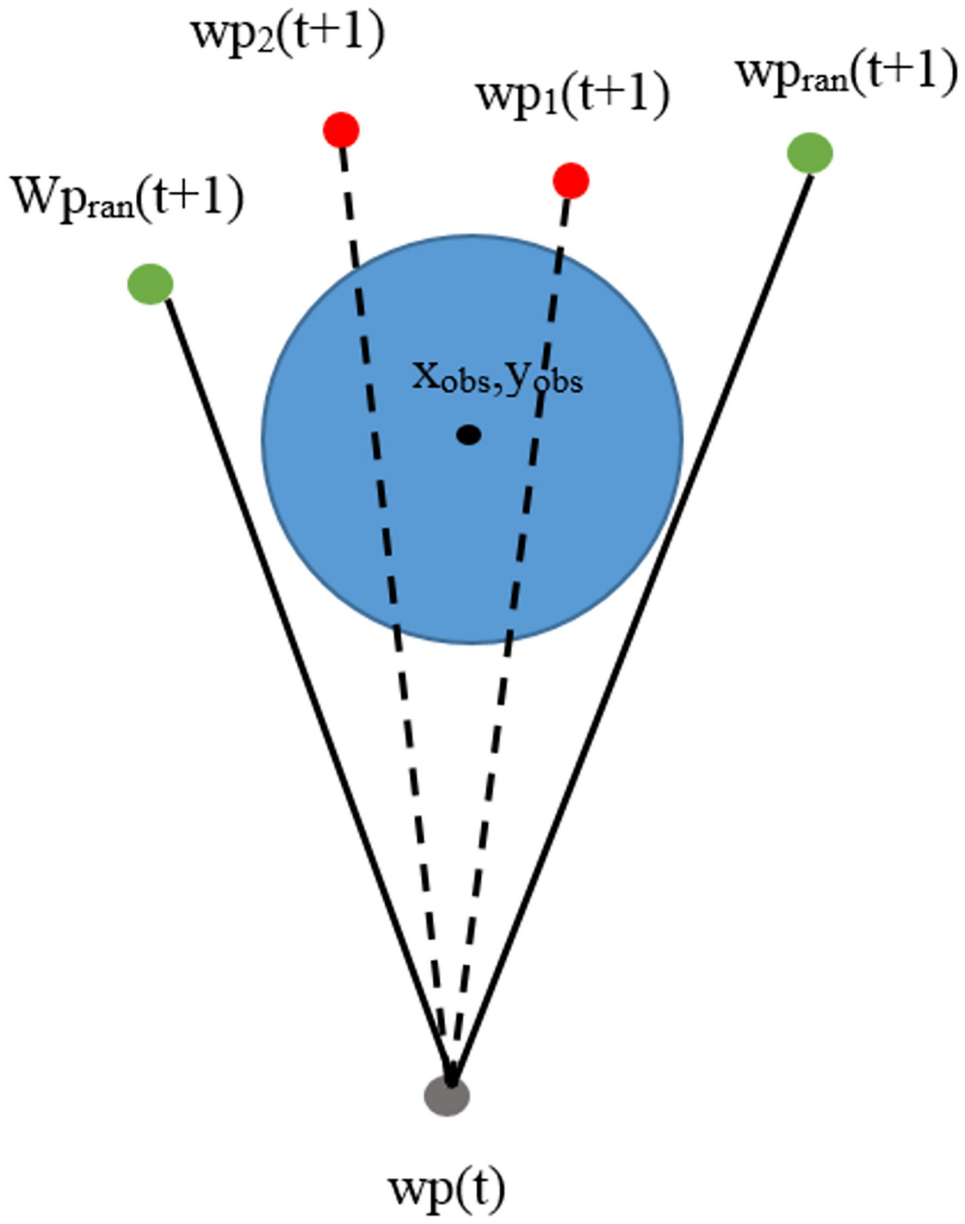
How to address infeasible waypoints when obstacles block paths.

In Fig 14, the blue circles represent obstacles in the environment. The center of the obstacles is determined to be in the position (*X*_*obs*_, *Y*_*obs*_). Current waypoints (*wp*(*t*)) is represented with a gray spot. Feasible waypoints are represented with 2 red spots, *wp*_1_(*t* + 1), and *wp*_2_(*t* + 1). They will be tested by drawing a straight line that connects current waypoints and feasible waypoints. If the straight line is drawn through the areas occupied by obstacles, they are infeasible waypoints, and paths cannot be connected between the current waypoint to their positions. Fig 15 shows how this problem is addressed.

In Fig 15, it is noted that feasible waypoints *wp*_1_(*t* + 1) and *wp*_2_(*t* + 1) are blocked by obstacles, making them infeasible waypoints, and paths cannot be connected from the current waypoint (*wp*(*t*)) to their positions. In this study, the proposed method randomly selects new possible movement points *wp*_*ran*_(*t* + 1) represented with green spots. The randomly selected points are tested by connecting a part of a straight line from the current waypoint to the randomly selected points. If the straight line does not pass through the areas occupied by obstacles, these points are feasible waypoints, and paths can be connected from the current waypoint to them. Otherwise, feasible waypoints shall be randomly selected again.

### Obstacle avoidance

When a robot moves to a different environment, it needs to know the positions of the obstacles. When it moves closer to obstacles, it must be able to avoid them. In this study, the robot uses a sensor to detect and identify the positions of obstacles in the environment. The characteristics of the sensors are as follows: It can detect (scan) obstacles in 360 degrees, It can detect obstacles within 6 meters, and Its scanning frequency is 10*H*_*z*_ (600 rmp).

The operating process of the sensor comprises two steps. The first step is called an acquisition process. It is a process of collecting environmental data surrounding the robot. During this step, the sensor rotates around itself and emits light from the emitter. Next, a timer determines the time that the light travels to objects and reflects back to the receiver. The distance of objects can be calculated from Eq (19). This technique is called time of flight (TOF) [38].

$$\begin{array}{c} s=c*t \end{array}$$ (19)

where *s* is the distance between obstacles and the sensor, *c* is the velocity of the light, and *t* is the duration that the light travels from the emitter and reflects back to the receiver.

**Fig 16:**
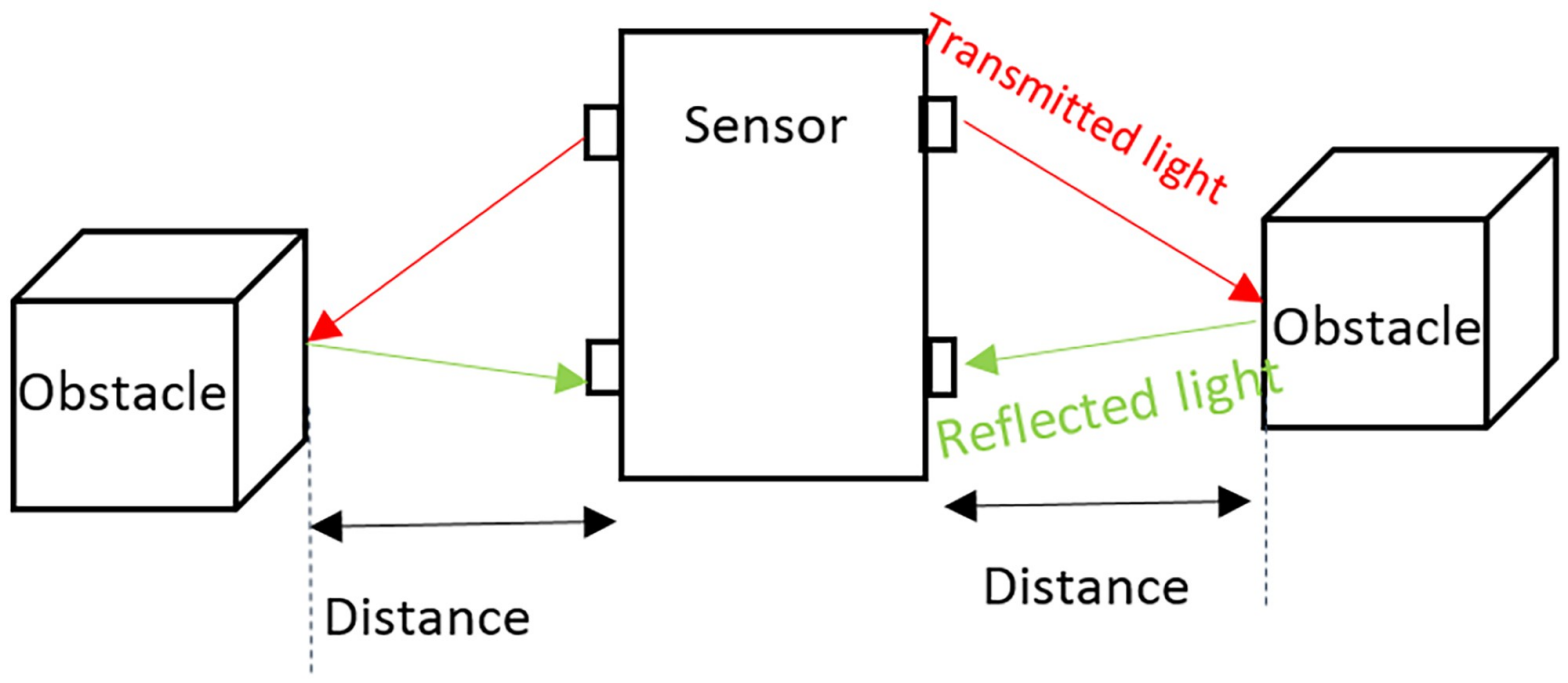
TOF operating process.

In Fig 16, when the sensor starts working, it will rotate around itself clockwise and emit sensor light from the emitter. When the light strikes the surface of obstacles, it will reflect to the receiver and record the time that the light is emitted until reflected back to the receiver. The recorded time is used to calculate the distance between the sensor and obstacles according to Eq (19).

**Fig 17:**
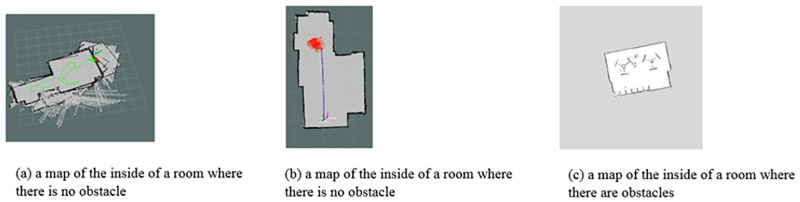
Examples of the maps obtained from the sensor.

**Fig 18:**
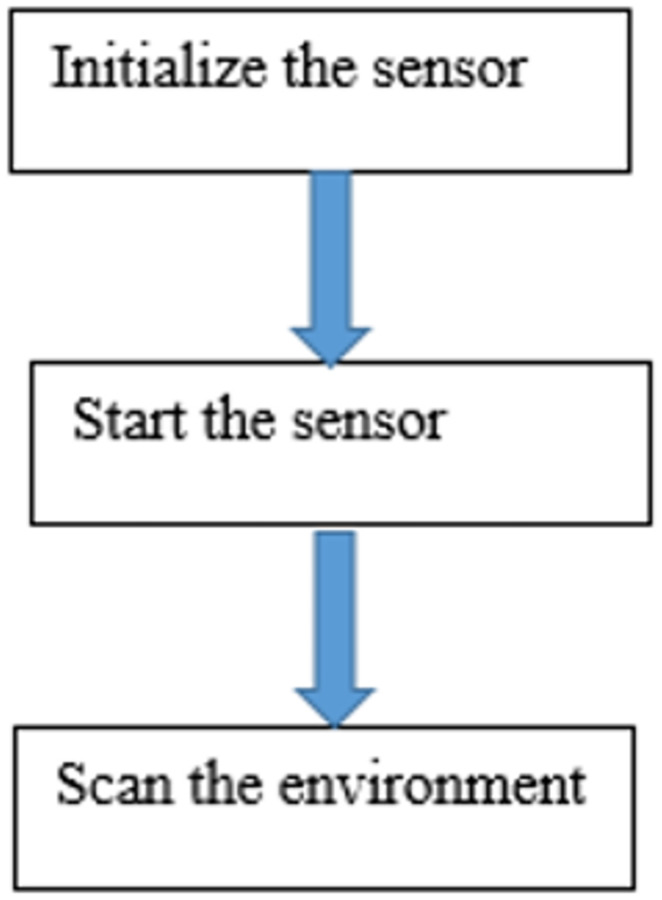
The sensor flowchart.

In Fig 17(a)–17(c) shows examples of the maps obtained from the sensor. Fig 17(a) and 17(b) shows maps of the inside of a room where there is no obstacle, while Fig 17(c) shows a map of the inside of a room where there are obstacles. Based on the environmental data collection step, the sensor will scan the environment continuously with the following working procedures:

As you can see from the Fig 18, The first operating procedure is an initialize the sensor. In this procedure many parameters are defined. First of all is set the port number. Serial port name to which sensor is connected. After that is set baudrate. Baudrate for serial connection (the default is 115200). Next is set timeout. Serial port connection timeout in seconds (the default is 1). Next is set logger to monitor the sensor work. _init_(port, baudrate = 115200, timeout = 1, logger = None). Next, connects to the serial port with the name self.port. If it was connected to another serial port disconnects from it first. The second operating procedure is start the sensor. In this step we use the command start_motor() to start the sensor. Next, Get device information with command get_info(). Next is get sensor health state, get device health state. When the core system detects some potential risk that may cause hardware failure in the future, the returned status value will be ‘Warning’. But sensor can still work as normal. When sensor is in the Protection Stop state, the returned status value will be ‘Error’. In case of warning or error statuses non-zero error code will be returned. Then, Maximum number of measurements to be stored inside the buffer. Once number exceeds this limit buffer will be emptied out. The last operating procedure is scan the environment. The first step in this procedure is iterate over measurements. Note that consumer must be fast enough, otherwise data will be accumulated inside buffer and consumer will get data with increasing lag. Next is set flag of new scan. The value is true if measurement belongs to a new scan. Then, set reflected laser pulse strength. After that, set the measurement heading angle in degree unit [0, 360). Next step is set the measured object distance. Measured object distance related to the sensor’s rotation center. In millimeter unit. Set to 0 when measurement is invalid. Next is set the minimum number of measurements. Minimum number of measurments in the scan for it to be yielded. After that is Iterate over scan with command iter_scans(max_buf_meas = 500, min_len = 5). The last is List of the measurements. Each measurement is tuple with following format: (quality, angle, distance).

**Fig 19:**
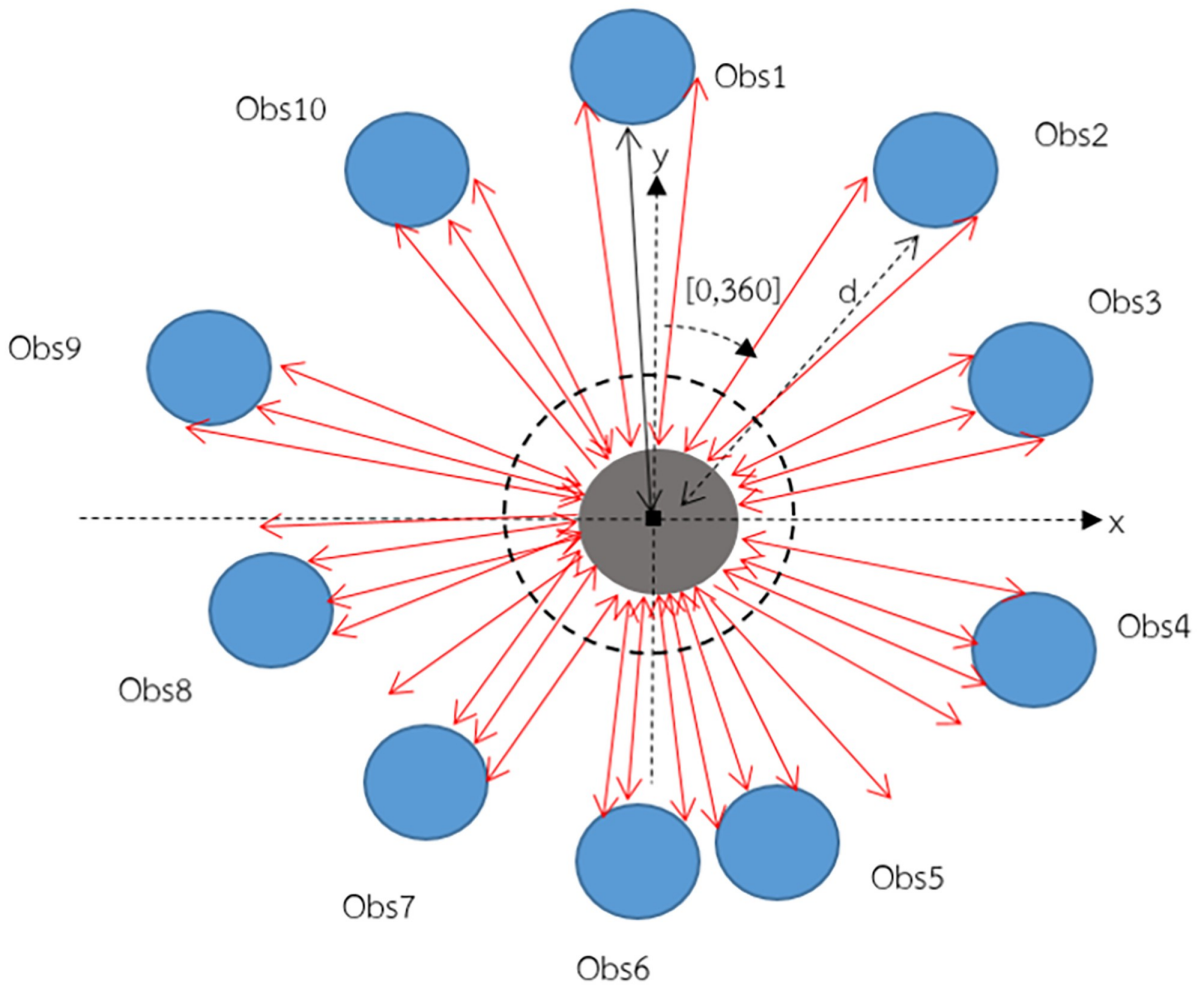
Determination of obstacle positions using sensor.

The sensor can also utilize data extraction. The sensor will search for obstacles having the smallest distance from the robot by checking distance values. Then, it will search for those obstacles from angle values to obtain an angle value at which the obstacles act on the robot. Determination of the obstacle positions for the sensor follows the left-hand rule of the coordinate system [38], as seen in Fig 19.

Fig 19 shows the simulation of detecting obstacles surrounding the robot using a sensor that follows the left-hand rule of the coordinate system. The robot is represented with a gray circle. The sensor is installed on the robot. The sensor is represented with a black square. Blue circles represent obstacles in the environment. In Fig 19, there are 10 obstacles (obs1-obs10). The horizontal dashed line and vertical dashed line represent plane axes in a two-dimensional coordinate plane (x,y) to identify the positions of obstacles on the two-dimensional plane. When the sensor starts to work, it will rotate around itself clockwise. The degree of rotation allows it to rotate all the way around. Its degree of rotation is in the range of 0-360 degrees. While the sensor rotates around itself, it will emit light from the emitter. When the sensor light strikes the surface of obstacles, the light will reflect back to the sensor receiver (represented with a two-directional arrow sign). The sensor uses time data of the sensor light traveling from and to calculate the distance between the sensor and obstacles according to the TOF method. For example, the sensor detects that obs2 has a d meter distance. The sensor can detect obstacles at 360 degrees.

In the previous decade, multi-objective particle swarm optimization (MOPSO) was introduced, by which optimization can be performed for more than one conflicting objectives simultaneously. The MOPSO was proposed to optimize more than one objective functions. In MOPSO instead of a single solution a set of solutions are determined, also called pareto optimal set [39]. The differences between the MOPSO and the proposed algorithm (MOEPSO) are as follows. Firstly, the solution given by MOPSO are a set of solutions called pareto optimal set whereas the answer given by MOEPSO is a single one, at which waypoint all 3 criteria of path search are met. Secondly, changing of the weight value. In MOPSO, the weighted value of movement (*w*), the weighted value for particle-based learning (*C*_1_) and the weighted value for group-based learning (*C*_2_) are constant result in getting trapped in a local optimal solution. On the other hand, in the proposed algorithm these values are adapted to change every iteration according to Eqs (15)–(17). In addition, a weight adjustment method is modified for the movement of particles in each iteration to increase the chance of finding the best fit solution according to Eq (18). Lastly, the proposed algorithm considers the degree of mutation, crossover, and selection to improve the efficiency of each particle.

## Experimental design

The simulation of the path planning algorithm are shown in two models. In the first one, the efficiency of the algorithm is tested in an environment with static obstacles. In the second model, the efficiency of the algorithm is tested in an environment with dynamic obstacles. All experiments are achieved the solutions after executing the algorithm using MATLAB R2017b programming language. The MATLAB codes are run on a computer system 2.60GHz CPU and 8 GB RAM. Parameter values in the experiment and environmental are detailed in this section.

### Parameter setting

Parameter values in the experiment for the MOEPSO algorithm are shown in Table 1.

**Table 1:** Parameter for MOEPSO algorithm.

Parameter	Value
Number of iteration	100
Number of particles	50
*C*_1_	1.5
*C*_2_	1.5
$C_{1}^{Min}$	0.1
$C_{1}^{Max}$	2.05
$C_{2}^{Min}$	0.1
$C_{2}^{Max}$	2.05
$K_{C_{1}}$	0.4
$K_{C_{2}}$	0.4
*f*_*min*_	0.2
*f*_*max*_	0.7
*w*	1
*K*_*w*_	0.4

Table 1 shows the parameter of MOEPSO algorithm. The number of iterations is set to 100 iterations. The number of particles is set to 50 particles. The weighted value for particle-based learning (*C*_1_) is set to 1.5. The weighted value for group-based learning (*C*_2_) is set to 1.5. The minimum weighted value of particle-based learning ($C_{1}^{Min}$) is set to 0.1. The maximum weighted value of particle-based learning ($C_{1}^{Max}$) is set to 2.05. The minimum weighted value for group-based learning ($C_{2}^{Min}$) is set to 0.1. value for group-based learning ($C_{2}^{Max}$) is set to 2.05. The factor value of the weighted value for particle-based learning ($K_{C_{1}}$) is set to 0.4. The factor value of the weighted value for group-based learning ($K_{C_{2}}$) is set to 0.4. The *f*_*min*_ is set to 0.2. The *f*_*max*_ is set to 0.7. The weighted value of movement (*w*) is set to 1. The factor value of the weighted value of movement (*K*_*w*_) is set to 0.4.

In the PSO algorithm, the three coefficient parameters are w, *C*_1_, and *C*_2_ that are held at constant values during the search process for all iterations. However, dynamic adjustment of these coefficient parameters according to the number of iteration is expected to achieve better results. At the beginning of search, a large *w* contributes to more effective exploration at global level, and at the later iterations, the exploitation at local search can be greatly enhanced by small inertia weight. The cognitive weight *C*_1_ signifies the affection of personal best experience. At the beginning of search, this coefficient should be large to enhance the exploration. But, as the final iterations are approached, it should be smaller to improve exploitation. This process implies that at the beginning of search, a particle position relies more on the past individual experience instead of the best position of the whole swarm, while the opposite occurs towards the end. The social weight *C*_2_ applies the affection of best position of the whole swarm. To prevent a particle from getting trapped in a local optimal point, the influence of this parameter should be enhanced according to the increasing iterations. At beginning, this parameter should have little influence on the particle position. While at later iterations, this parameter should be large in order to enhance the social communication between the particle and swarm. All three parameters will be adjusted according to Eqs (15)–(17).

### Path planning in static environment (5 obstacles)

This section examines the path planning algorithm in a static environment with 5 obstacles. A starting point is set at position (0,0), a destination is set at position (10,10), and the radius of the robot is 0.5 m. The determined positions and radius of each obstacle are shown in Table 2. Table 2 shows details for determining the radius and positions of obstacles in a static environment (5 obstacles): Obstacle 1 has a 0.5 meter radius and is at the position (2,2.3), obstacle 2 has a 0.8 meter radius and is at the position (5,4), obstacle 3 has a 1.2 meter radius and is at the position (8,2), obstacle 4 has a 1 meter radius and is at the position (7.7,7) and obstacle 5 has a 0.7 meter radius and is at the position (3,8.3).

**Table 2:** The obstacle data in the static environment (5 obstacles).

Obstacle sequence	Radius	Positions
1	0.5	(2,2.3)
2	0.8	(5,4)
3	1.2	(8,2)
4	1	(7.7,7)
5	0.7	(3,8.3)

### Path planning in static environment (4 obstacles)

This section shows path planning algorithms in a static environment with 4 obstacles. A starting point is determined at position (0,0), a destination is determined at position (10,10), and the radius of the robot is 0.5 m. The positions of each obstacle are determined as shown in Table 3. Table 3 shows the determination of the radius and position of each obstacle with the following details: Obstacle 1 has a radius of 1 meter and is at the position (4,1.5), obstacle 2 has a radius of 1 meter and is at the position (2,3.5), obstacle 3 has a radius of 2 meters and is at the position (7.5,6) and obstacle 4 has a radius of 1.5 meters and is at the position (3.5,7.5).

**Table 3:** The obstacle data in the static environment (4 obstacles).

Obstacle sequence	Radius	Positions
1	1	(4,1.5)
2	1	(2,3.5)
3	2	(7.5,6)
4	1.5	(3.5,7.5)

### Path planning in static environment (6 obstacles)

This section shows path planning algorithms in environments with 6 static obstacles. A starting point is determined at position (0,0), the destination is at position (10,10), and the radius of the robot is 0.5 m. The positions and radius of each obstacle are determined as shown in Table 4. Table 4 shows the determination of the radius and positions of each obstacle used in the path planning experiment in a static environment (6 obstacles). The details are as follow: Obstacle 1 has a radius of 0.8 meter and is at the position (2,1.5), obstacle 2 has a radius of 1 meter and is at the position (1,3.5), obstacle 3 has a radius of 0.9 meter and is at the position 5,1), obstacle 4 has a radius of 0.8 meter and is at the position (3.5,7.5), obstacle 5 has a radius of 1.7 meters and is at the position (5,6.2), and obstacle 6 has a radius of 0.9 meter and is at the position (9,4).

**Table 4:** The obstacle data in the static environment (6 obstacles).

Obstacle sequence	Radius	Positions
1	0.8	(2,1.5)
2	1	(1,3.5)
3	0.9	(5,1)
4	0.8	(3.5,7.5)
5	1.7	(5,6.2)
6	0.9	(9,4)

### Path planning in dynamic environment

In this section, path planning in a dynamic environment with moving obstacles is simulated. Obstacles in a dynamic environment can move in 2 models: linear movement obstacles and nonlinear movement obstacles, as mentioned earlier in section of obstacle movement. Obstacles moving in a straight line possess the characteristics shown in Table 5. Table 5 shows the characteristics of obstacles that move in a straight line. Obstacle 1 has a radius of 0.3 meters and is at positions 7.5 and 2.1, and its velocity of movement is equal to 0.16 m/s with a 70 degree direction of movement. Obstacle 2 has a radius of 0.3 meters and is at position (5.1,8.3) its velocity of movement is equal to 0.13 m/s with a 0 degree direction of movement. Furthermore, in the dynamic environment, there are nonlinear movement obstacles. Their characteristics are shown in Table 6.

**Table 5:** The characteristic of linear movement obstacles.

Obstacle sequence	Radius	Starting point	Speed (m/s)	Direction (deg)
1	0.3	(7.5,2.1)	0.16	70
2	0.3	(5.1,8.3)	0.13	0

**Table 6:** The characteristic of nonlinear movement obstacles.

Obstacle sequence	Radius	Starting point
3	0.3	(6,5)
4	0.3	(4,5)
5	0.3	(5,7.5)
6	0.3	(5,2.5)

In Table 6, the details are as follows: obstacle 3 has a starting point at position (6,5), and the radius is 0.3 meters. Obstacle 4 has a starting point at position (4,5), and the radius is 0.3 meters. Obstacle 5 has a starting point at the position (5,7.5) and the radius is 0.3 meter, and obstacle 6 has a starting point at the position (5,2.5) and the radius is 0.3 meter.

## Result and discussion

In this section, the simulation results of the proposed path planning algorithm in each environment are detailed. First of all, Path planning results in the static environment are detailed. After that, path planning results in the dynamic environment are shown. At the end of this section, we will discuss about the results.

### The result of path planning in static environment (5 obstacles)

In the path planning experiment in a static environment (5 obstacles), the algorithm was ran 10 times. The shortest path and time spent on the data processing are shown and compared to previous studies [12] in Tables 7 and 8.

**Table 7:** Path planning results in the static environment (5 obstacles).

No.	MFB [12]			MOEPSO		
	Path length	Fitness	Time (min)	Path length	Fitness	Time (min)
1	**14.7930**	**0.0676**	**0.5530**	14.6313	0.1417	0.1433
2	14.8112	0.0675	0.7384	14.7437	0.1389	0.2603
3	14.7930	0.0676	0.7050	14.8239	0.1337	0.3125
4	14.8112	0.0675	0.8844	14.7956	0.1234	0.3730
5	14.8525	0.0673	1.2217	**14.5989**	**0.1366**	**0.3448**
6	14.8028	0.0676	0.7457	14.7315	0.1401	0.3685
7	14.8798	0.0672	0.8554	14.6254	0.1422	0.3139
8	14.8051	0.0675	0.8571	15.1456	0.1324	0.2959
9	14.7930	0.0676	0.6038	14.8952	0.1433	0.2580
10	14.8112	0.0675	0.7717	14.7086	0.1433	0.2715
Average	14.8153	0.0675	0.7936	14.7700	0.1376	0.2942

**Table 8:** Path planning results in the static environment (5 obstacles) Cont.

No.	Hybrid PSO-MFB [12]			MOEPSO		
	Path length	Fitness	Time (min)	Path length	Fitness	Time (min)
1	14.7860	0.0676	3.4253	14.6313	0.1417	0.1433
2	14.7953	0.0676	3.1892	14.7437	0.1389	0.2603
3	14.7960	0.0676	3.7041	14.8239	0.1337	0.3125
4	14.8083	0.0675	3.3686	14.7956	0.1234	0.3730
5	14.7909	0.0676	3.3162	**14.5989**	**0.1366**	**0.3448**
6	**14.7785**	**0.0677**	**3.4836**	14.7315	0.1401	0.3685
7	14.7915	0.0676	3.7204	14.6254	0.1422	0.3139
8	14.7876	0.0676	3.5218	15.1456	0.1324	0.2959
9	14.8023	0.0676	3.2010	14.8952	0.1433	0.2580
10	14.7921	0.0676	3.2472	14.7086	0.1433	0.2715
Average	14.7929	0.0676	3.4177	14.7700	0.1376	0.2942

In Tables 7 and 8, the proposed algorithm and algorithms investigated in previous research studies, i.e., the MFB algorithm and hybrid PSO-MFB algorithm, were tested, and the efficiency was compared. In this experiment, each algorithm was processed 10 times. The results obtained from each processing were divided into distance values from a starting point to a destination (path length), fitness values, and time spent processing (computational time). The experiment found that the path obtained from the MOEPSO algorithm was 14.5989 meters,. Based on the comparison, the path is shorter than the paths obtained from the hybrid PSO-MFB and MFB algorithms: 14.7785 meters and 14.7930 meters, respectively. In addition, the time spent processing the MOEPSO algorithm was 0.3448 minutes, which is less than the time spent processing the hybrid PSO-MFB algorithm and MFB algorithm, which is 3.4836 minutes and 0.5530 minutes, respectively. Moreover, the fitness values obtained from the MOEPSO algorithm was 0.1366, which is better than the fitness values obtained from the hybrid PSO-MFB algorithm and MFB algorithm, which is 0.0677 and 0.0676, respectively. Therefore, the findings from the experiment reveal that the proposed algorithm can determine the best path for the robot, as it is the shortest path with the least processing time and high fitness value.

**Fig 20:**
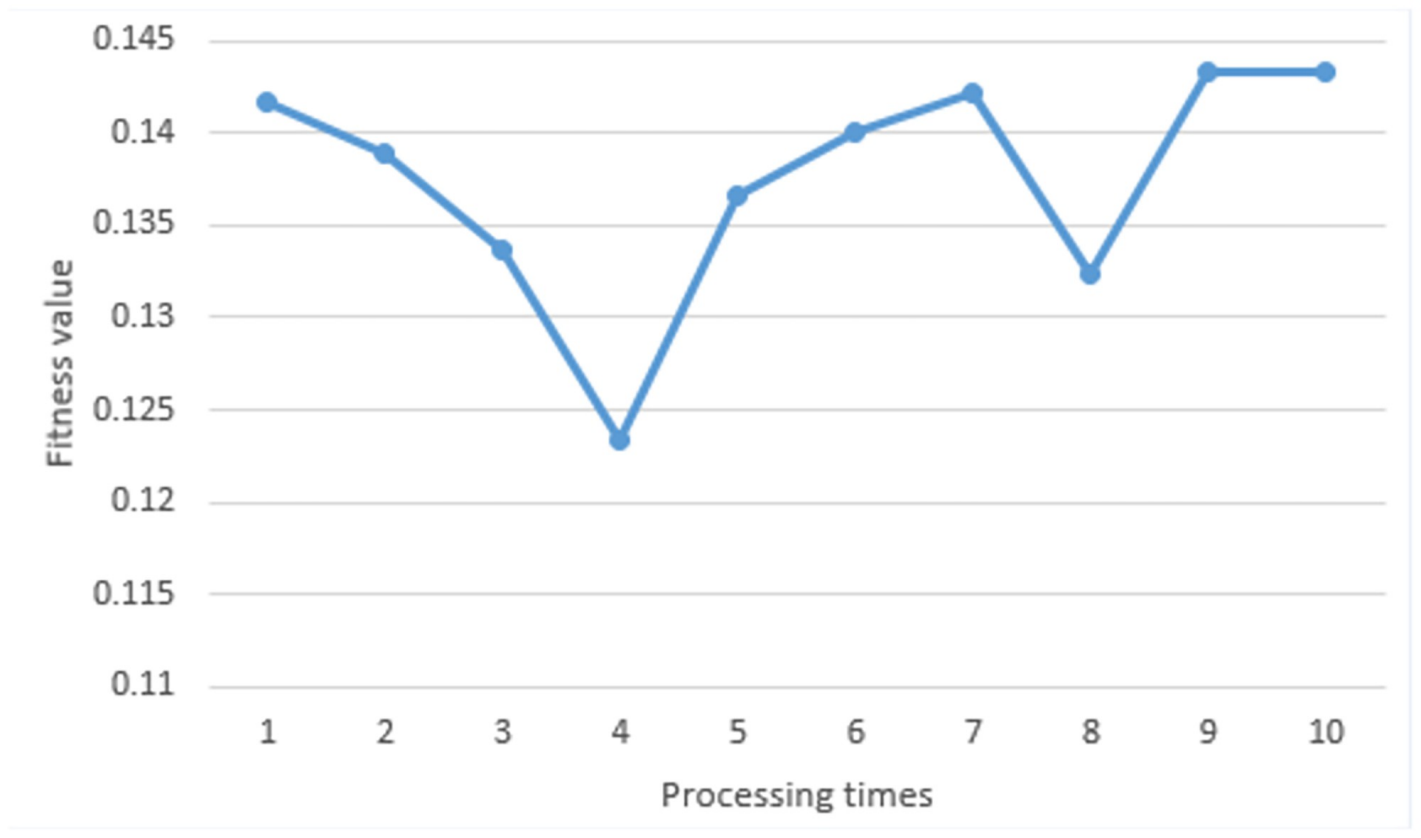
The fitness value obtained in a static environment (5 obstacles).

**Fig 21:**
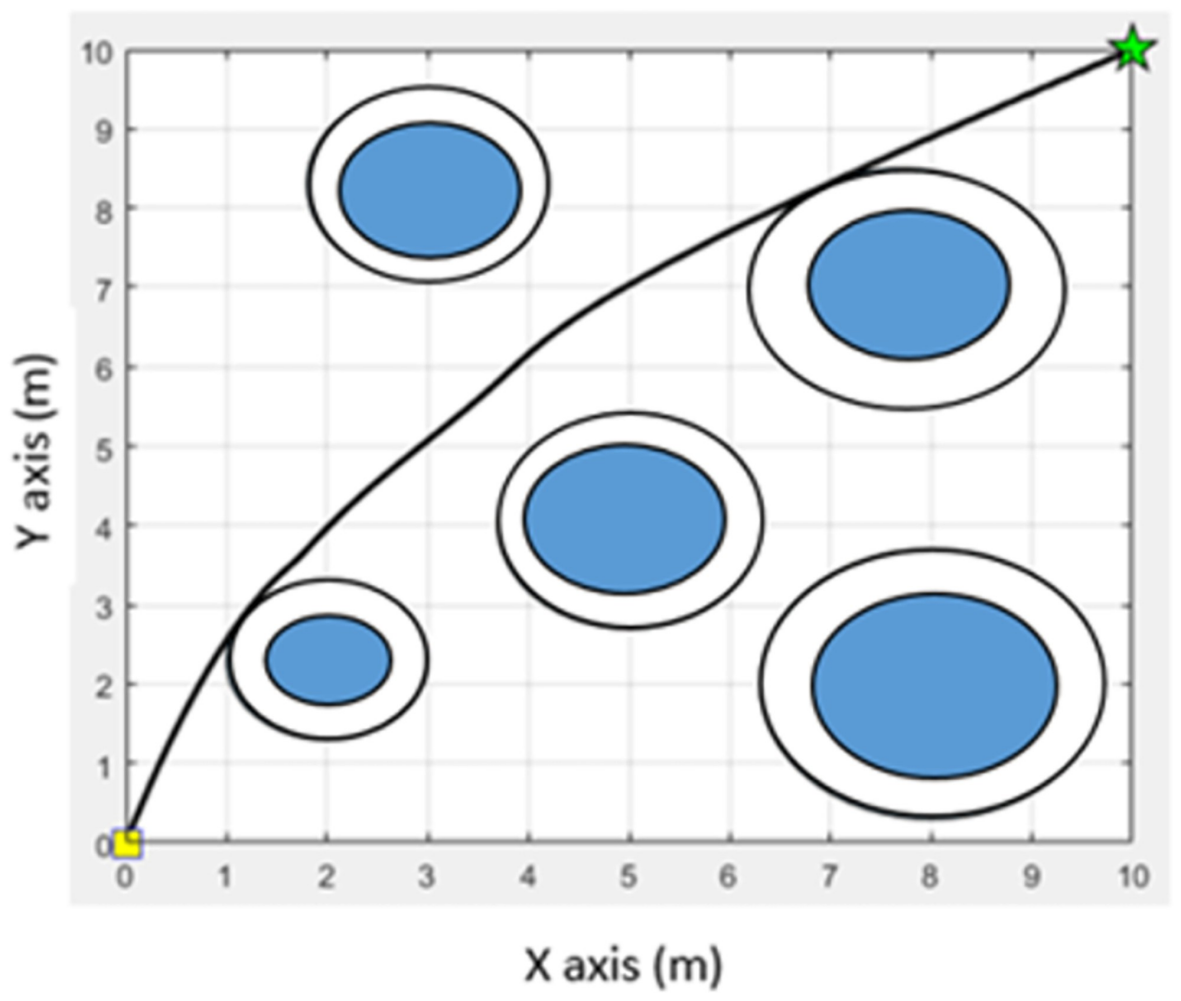
The path obtained from the MOEPSO algorithm in a static environment (5 obstacles).

Experimenting in a static environment with 5 obstacles, we ran the proposed algorithm 10 times. Fig 20 shows the fitness value obtained from each processing time. At the first time, the fitness value was 0.1417. At the second time, the fitness value was 0.1389. At the third time, the fitness value was 0.1337. At the fourth time, the fitness value was 0.1234. At the fifth time, the fitness value was 0.1363. At the sixth time, the fitness value was 0.1401. At the seventh time, the fitness value was 0.1422. At the eighth time, the fitness value was 0.1324. At the ninth time, the fitness value was 0.1433. At the last time, the fitness value was 0.1433. In each iteration, the particles with the best fitness value were chosen as the waypoint of path. These points were drawn as the optimal path as shown in Fig 21.

**Fig 22:**
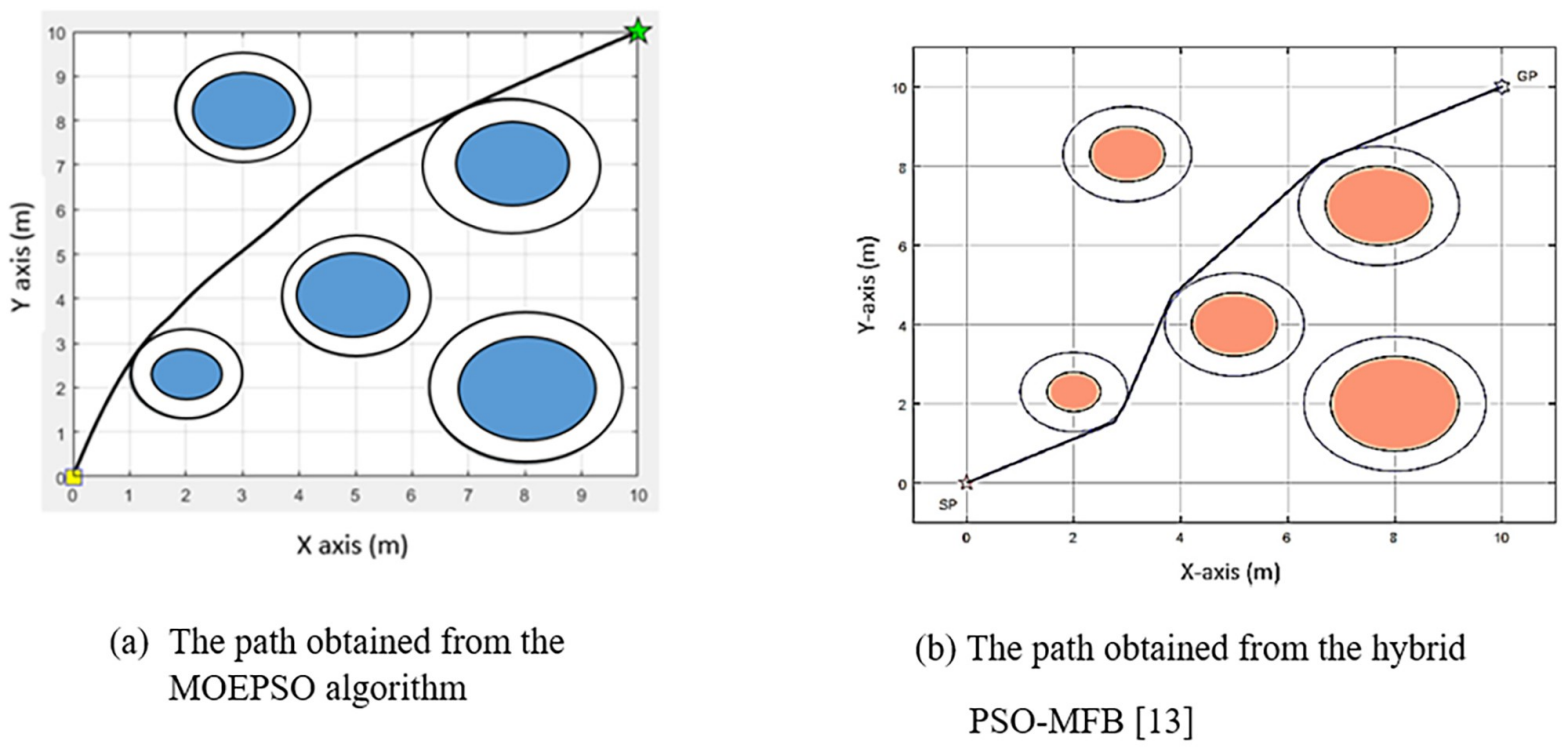
A comparison of the paths obtained from the MOEPSO and the hybrid PSO-MFB [12].

**Fig 23:**
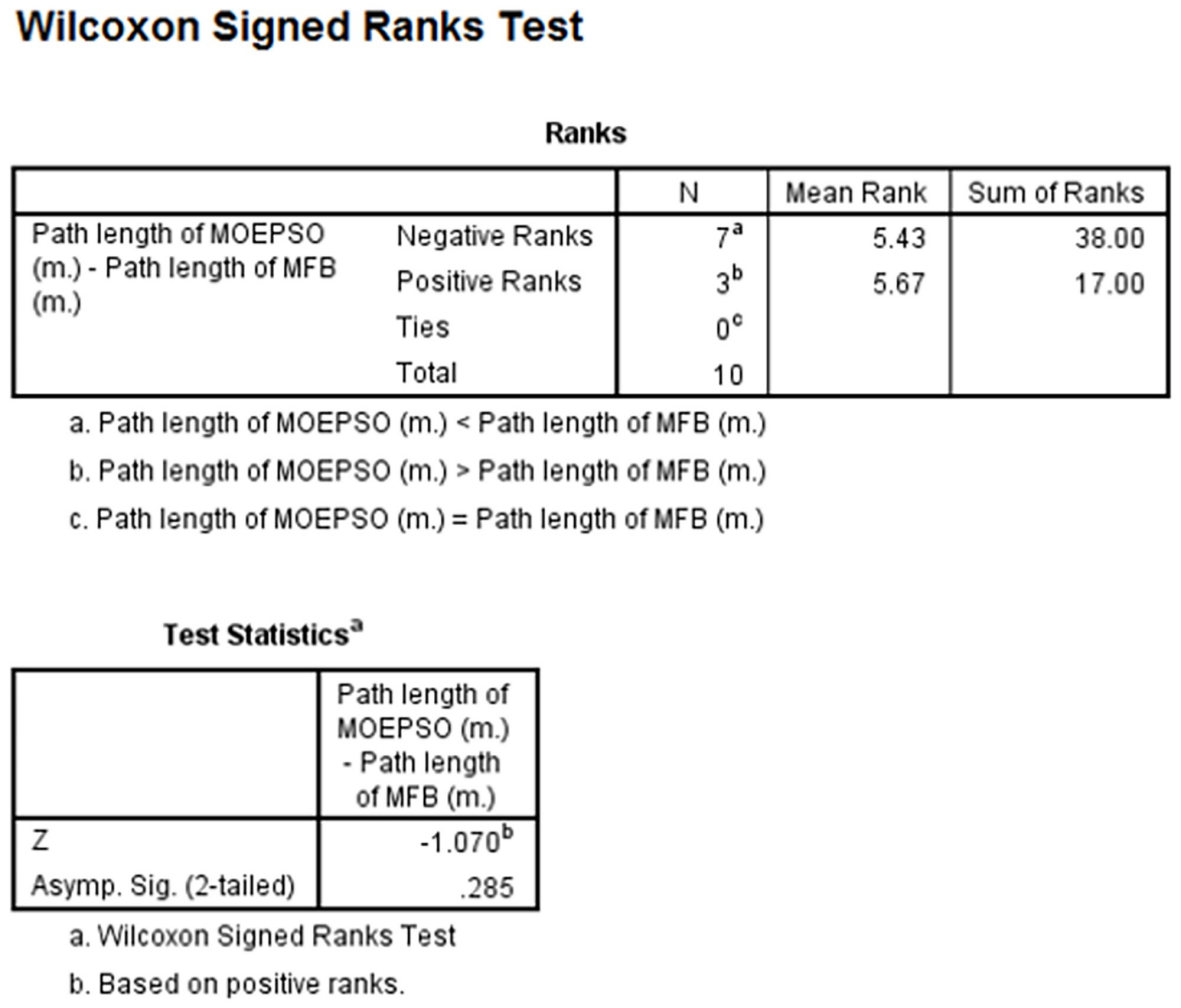
Wilcoxon signed-rank test path length of MOEPSO-path length of MFB.

Fig 22 shows a comparison of the paths obtained from the path planning experiment in a static environment with 5 obstacles. Fig 22(a) shows the path obtained from the MOEPSO algorithm, and Fig 22(b) shows the path obtained from the hybrid PSO-MFB algorithm. The path obtained from the MOEPSO algorithm has a smaller distance value from the starting point to the destination point than the path obtained from the hybrid PSO-MFB algorithm. Moreover, in this experiment The distance obtained from each algorithm was statistically tested with Wilcoxon signed-rank test. The results of statistical comparisons between path length obtained from MFB and MOEPSO are shown in Fig 23.

**Fig 24:**
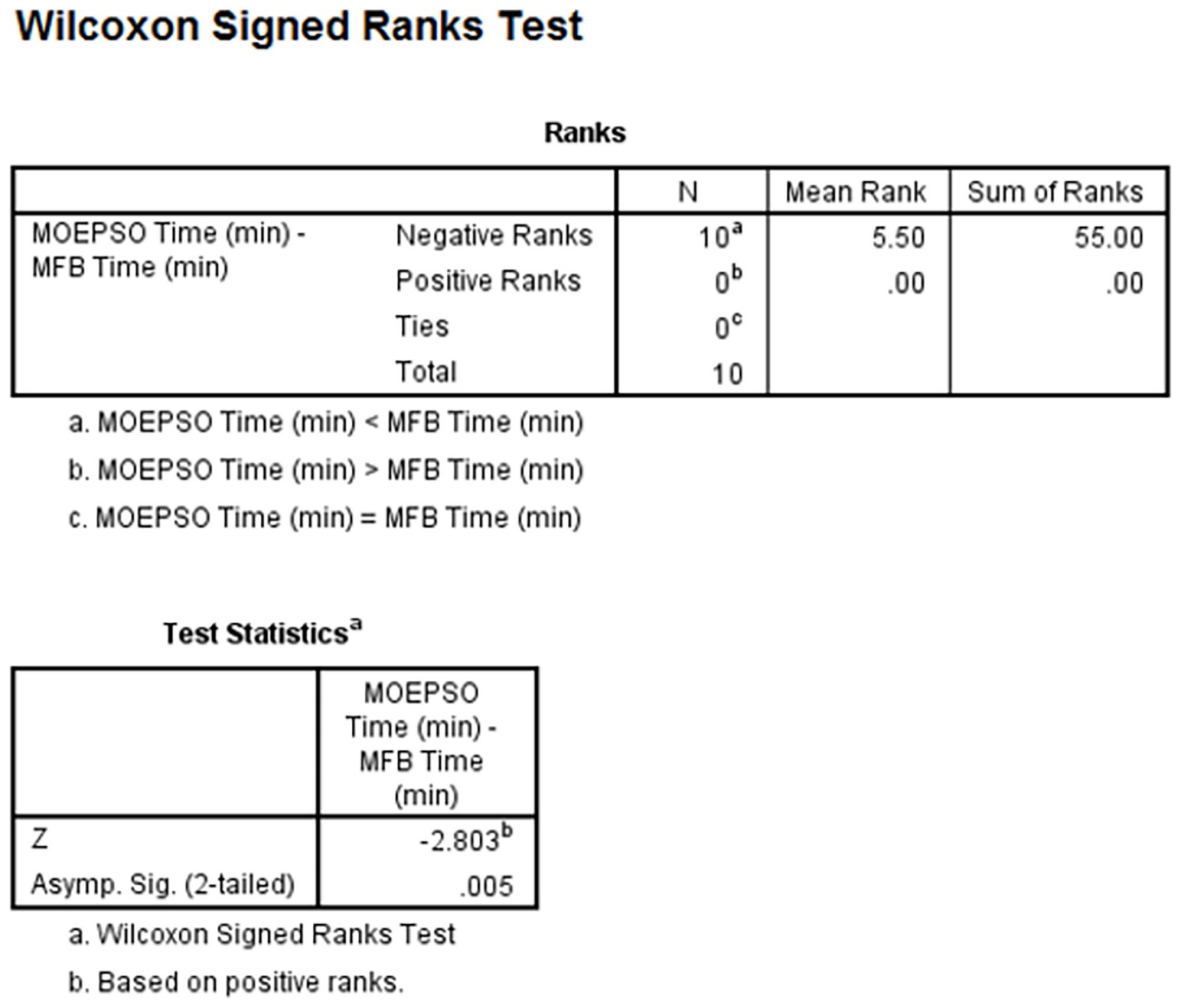
Wilcoxon signed-rank test processing time of MOEPSO-processing time of MFB.

As you can see from the figure, the number of Negative Ranks and Positive Ranks are equal to 7 and 3, respectively (column N). The sum of Negative Ranks and Positive Ranks are equal to 38 and 17, respectively (column Sum of Ranks). The negative ranks mean the path length obtained from MOEPSO is less than the path length obtained from MFB. The positive ranks mean the path obtained from MOEPSO is greater than rhe path obtained from MFB. In addition, there are no the data given the same path length. The statistical value used for the test, z-value, was given a value of -1.07, which is less than 0.05. We can therefore say that the path length obtained from MOEPSO was significantly reduced. The results of statistical comparisons between processing time of MFB and MOEPSO are shown in Fig 24.

**Fig 25:**
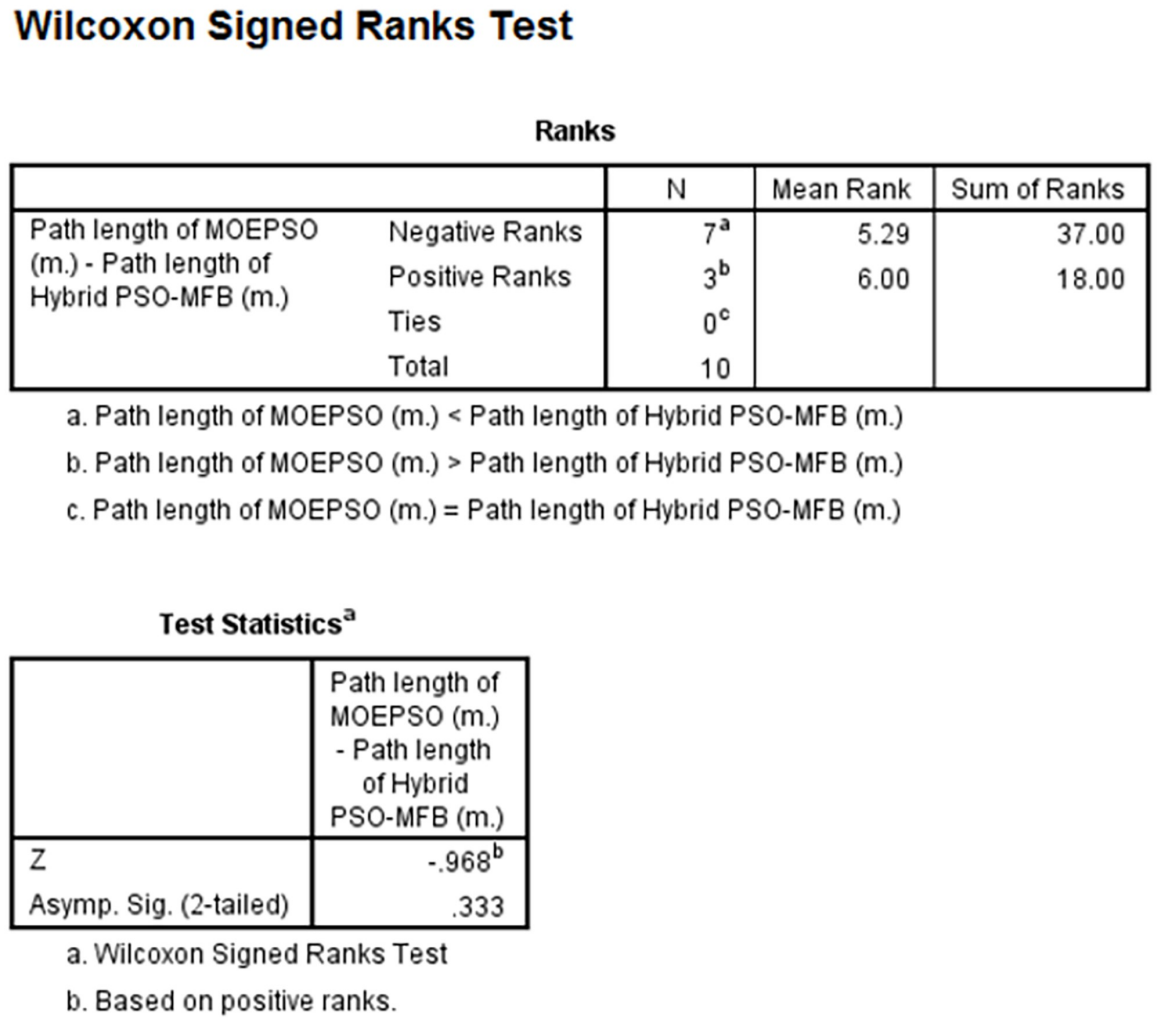
Wilcoxon signed-rank test path length of MOEPSO-path length of Hybrid PSO-MFB.

As you can see from the figure, the number of Negative Ranks and Positive Ranks are equal to 10 and 0, respectively (column N). The sum of Negative Ranks and Positive Ranks are equal to 55 and 0, respectively (column Sum of Ranks). The negative ranks mean the processing time of MOEPSO is less than the processing time of MFB. The positive ranks mean the processing time of MOEPSO is greater than the processing time of MFB. In addition, there are no the data given the same processing time. The statistical value used for the test, z-value, was given a value of -2.803, which is less than 0.05. We can therefore say that the processing time of MOEPSO was significantly reduced. The results of statistical comparisons between path length obtained from Hybrid PSO-MFB and MOEPSO are shown in Fig 25.

**Fig 26:**
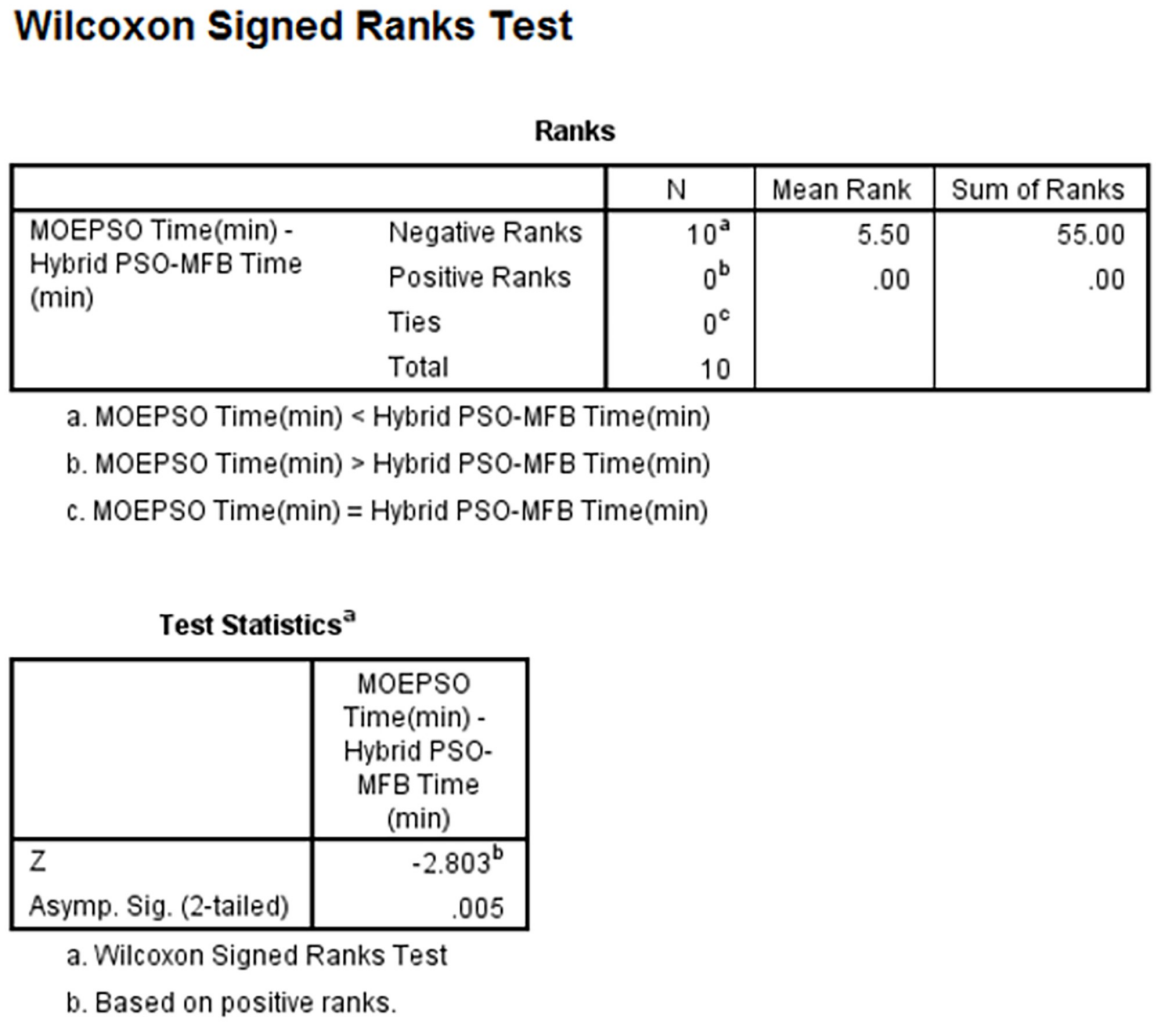
Wilcoxon signed-rank test processing time of MOEPSO-processing time of Hybrid PSO-MFB.

As you can see from the figure, the number of Negative Ranks and Positive Ranks are equal to 7 and 3, respectively (column N). The sum of Negative Ranks and Positive Ranks are equal to 37 and 18, respectively (column Sum of Ranks). The negative ranks mean the path length obtained from MOEPSO is less than the path length obtained from Hybrid PSO-MFB. The positive ranks mean the path obtained from MOEPSO is greater than the path obtained from Hybrid PSO-MFB. In addition, there are no the data given the same path length. The statistical value used for the test, z-value, was given a value of -1.07, which is less than 0.05. We can therefore say that the path length obtained from MOEPSO was significantly reduced. The results of statistical comparisons between processing time of Hybrid PSO-MFB and MOEPSO are shown in Fig 26.

As you can see from the figure, the number of Negative Ranks and Positive Ranks are equal to 10 and 0, respectively (column N). The sum of Negative Ranks and Positive Ranks are equal to 55 and 0, respectively (column Sum of Ranks). The negative ranks mean the processing time of MOEPSO is less than the processing time of Hybrid PSO-MFB. The positive ranks mean the processing time of MOEPSO is greater than the processing time of Hybrid PSO-MFB. In addition, there are no the data given the same processing time. The statistical value used for the test, z-value, was given a value of -2.803, which is less than 0.05. We can therefore say that the processing time of MOEPSO was significantly reduced.

### The result of path planning in static environment (4 obstacles)

In this experiment, the proposed algorithm was ran 10 times. The obtained path was compared to algorithms investigated in previous studies, i.e., the hybrid PSO-MFB algorithm [12], DABC algorithm [40], MAABC algorithm [40], GA algorithm [41] and BC algorithm [41]. Experimental results are shown and compared in Table 9.

**Table 9:** Path planning results in the static environment (4 obstacles).

Algorithm name	Path length (m)
MOEPSO	**14.3222**
Hybrid PSO-MFB [12]	14.3255
DABC [40]	14.3625
MAABC [40]	14.3371
GA [41]	14.5095
BC [41]	14.3802

From Table 9, it can be seen that the proposed algorithm was processed 10 times. The shortest path was shown and compared to the algorithms investigated in previous studies, i.e., the hybrid PSO-MFB algorithm, DABC algorithm, GA and BC algorithms. The experimental results indicate that the path obtained from the MOEPSO algorithm was 14.3222 meters. It is the shortest path compared to the paths obtained from the hybrid PSO-MFB, DABC, MAABC, GA and BC algorithms, which are 14.3255 meters, 14.3625 meters, 14.3371, 14.5095 meters and 14.3802 meters, respectively. Moreover, the fitness value obtained from the proposed algorithm was 0.1416.

**Fig 27:**
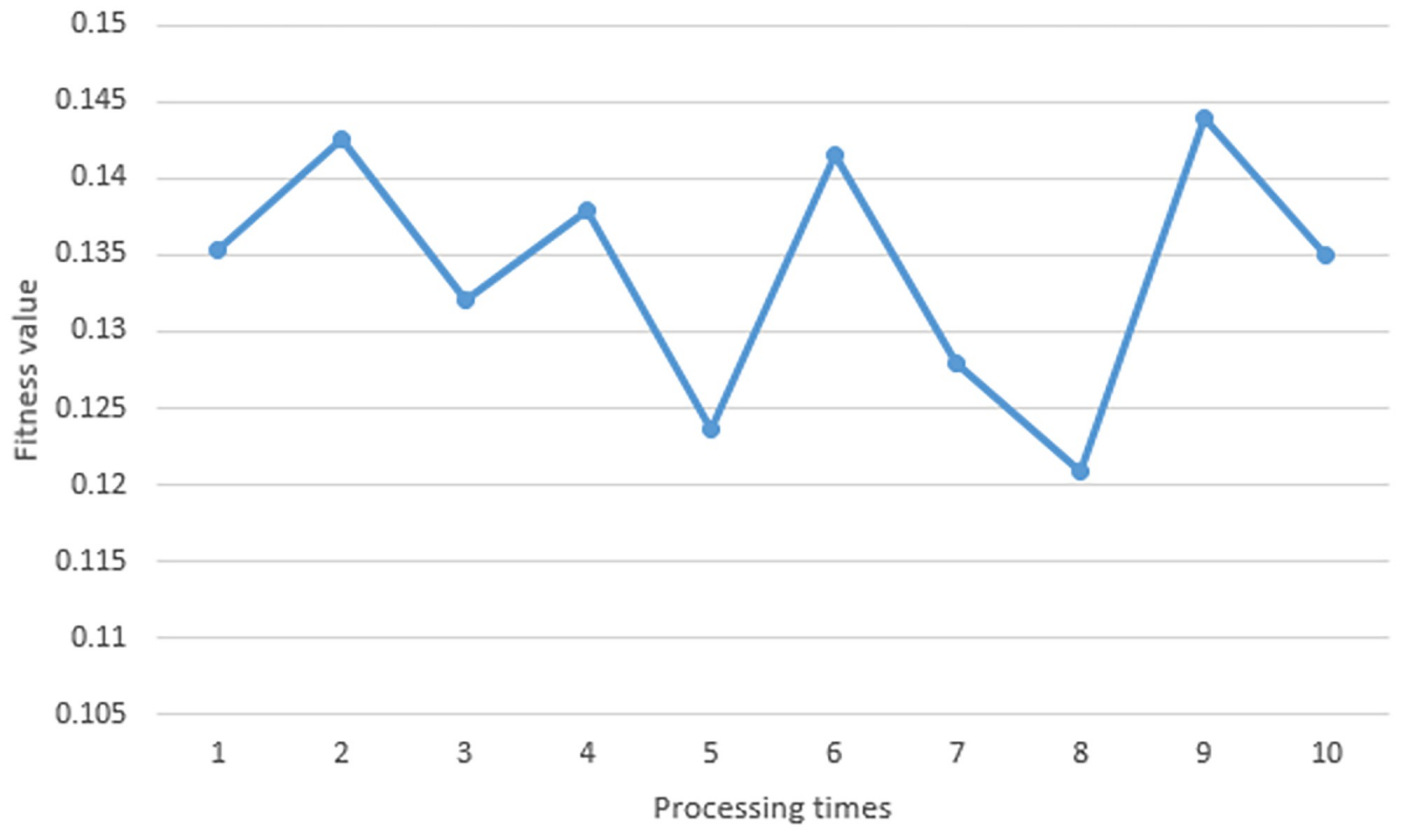
The fitness value obtained in a static environment (4 obstacles).

**Fig 28:**
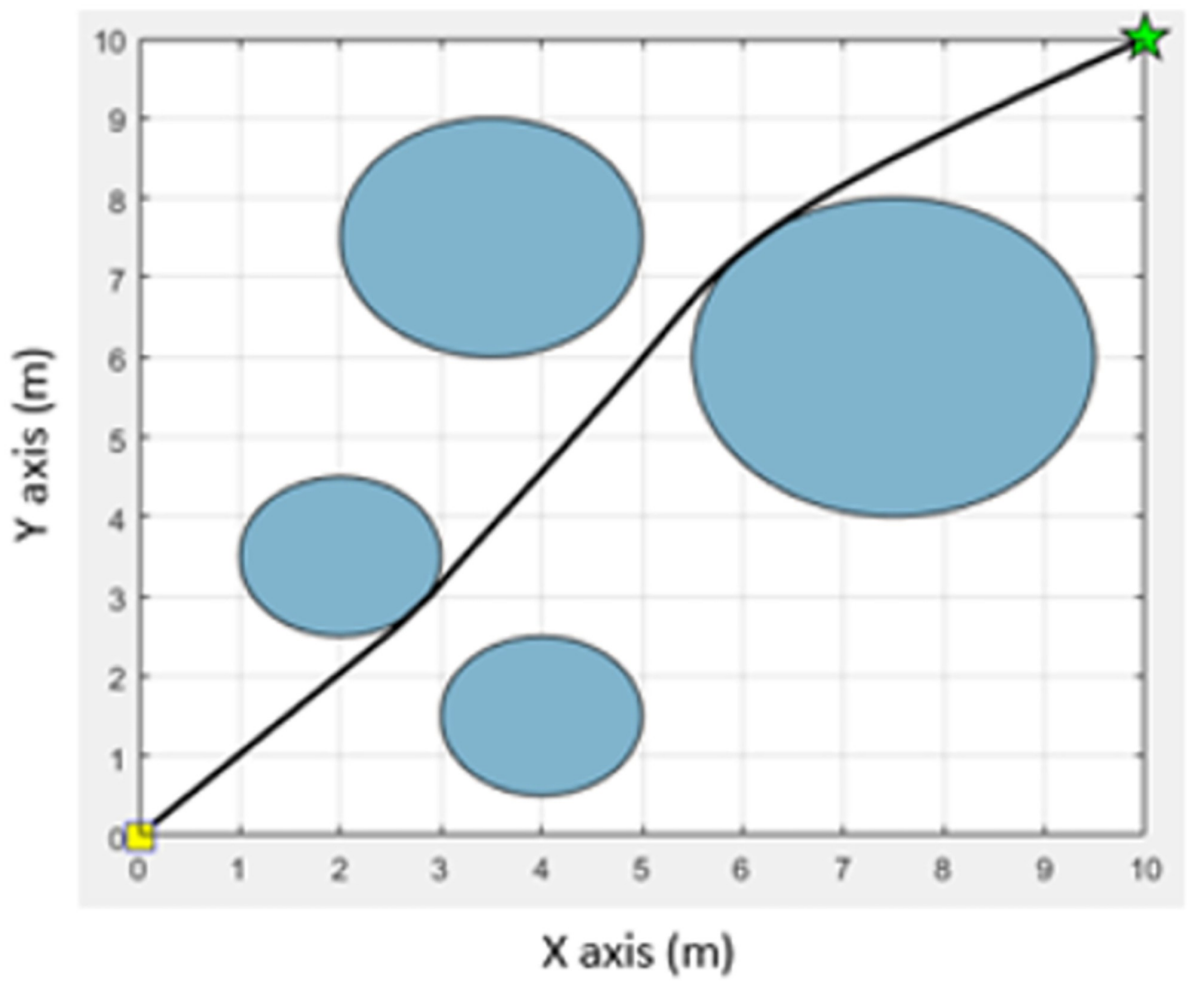
The path obtained from the MOEPSO algorithm in a static environment (4 obstacles).

Experimenting in a static environment with 4 obstacles, we ran the proposed algorithm 10 times. Fig 27 shows the fitness value obtained from each processing time. At the first time, the fitness value was 0.1353. At the second time, the fitness value was0.1426. At the third time, the fitness value was 0.1321. At the fourth time, the fitness value was 0.1379. At the fifth time, the fitness value was 0.1236. At the sixth time, the fitness value was 0.1416. At the seventh time, the fitness value was 0.1279. At the eighth time, the fitness value was 0.1209. At the ninth time, the fitness value was 0.1439. At the last time, the fitness value was 0.135. In each iteration, the particles with the best fitness value were chosen as the waypoint of path. These points are drawn as the optimal path as shown in Fig 28.

**Fig 29:**
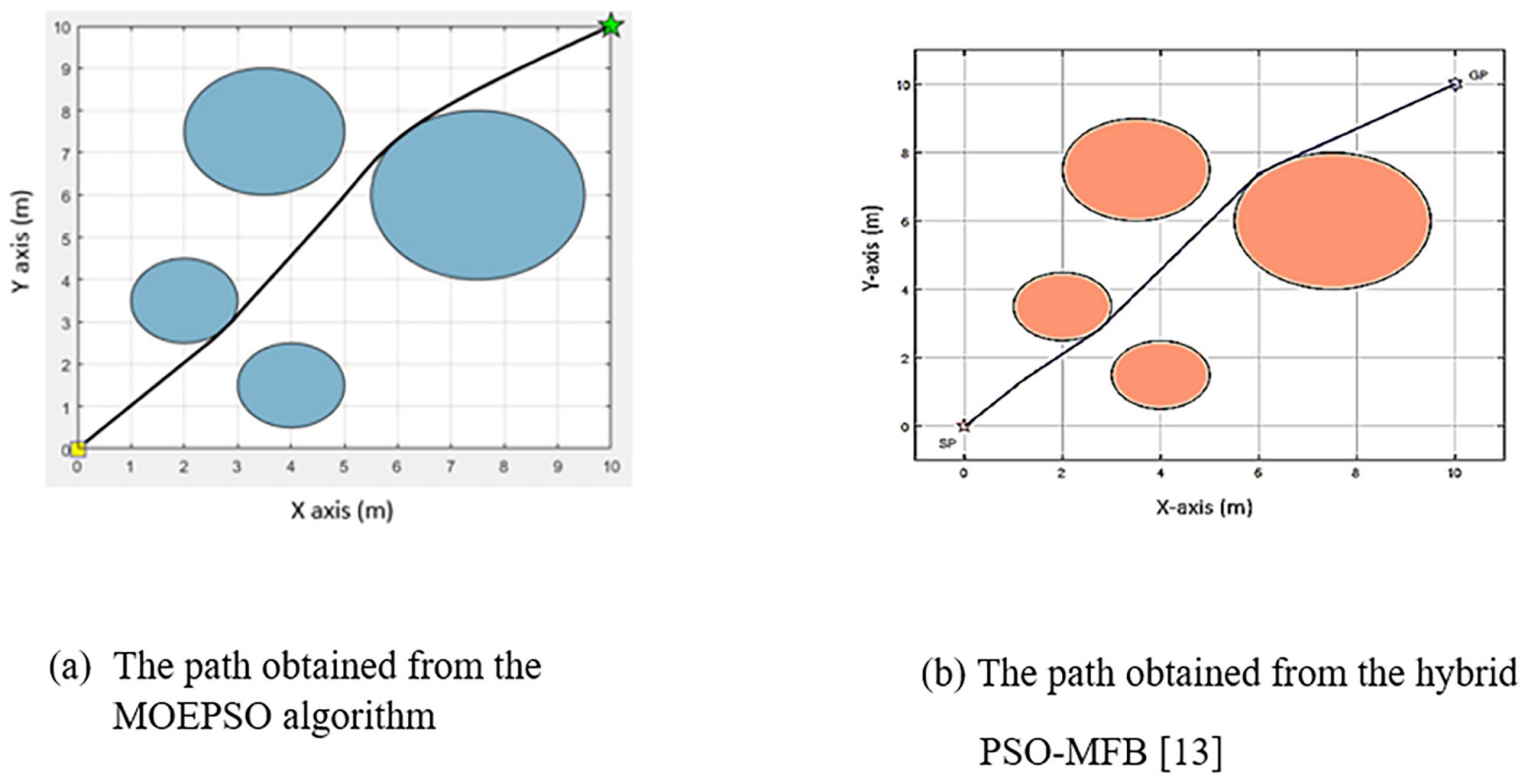
A comparison of the paths obtained from the MOEPSO and the hybrid PSO-MFB [12].

Fig 29 shows a comparison of the paths obtained from the path planning experiment in a static environment with 4 obstacles. Fig 29(a) shows the path obtained from the MOEPSO algorithm, and Fig 29(b) shows the path obtained from the hybrid PSO-MFB algorithm. It can be seen that the path obtained from MOEPSO has a smaller distance value from the starting point to destination point than the path obtained from the hybrid PSO-MFB algorithm.

### The result of path planning in static environment (6 obstacles)

In this experiment, the proposed algorithm was ran 10 times. The obtained shortest path is shown, and its performance is compared to the algorithms [12] investigated in previous studies, i.e., the hybrid PSO-MFB algorithm [12], standard ABC algorithm [42], DABC algorithm and MAABC algorithm [40]. A comparison of working efficiency is shown in Table 10. In Table 10, it can be seen that the proposed algorithm was processed 10 times. The shortest path was shown and compared to the algorithms investigated in previous studies, i.e., the hybrid PSO-MFB algorithm, standard ABC algorithm, DABC algorithm, and MAABC algorithm. Since the environment in the ABC algorithm test is 100 × 100 meters, the size of the environment is reduced 10 times to 10 × 10 meters to ensure that the experimental result comparison is conducted in a fair manner. The results from the experiment reveal that the path obtained from MOEPSO was 14.4743 meters. The obtained path is the shortest compared to the paths obtained from the hybrid PSO-MFB, standard ABC, DABC, and MAABC algorithms, which are 14.6384 meters, 14.8821 meters, 14.7422 meters, and 14.7163 meters, respectively. Moreover, the fitness value obtained from the proposed algorithm was 0.1312.

**Table 10:** Path planning results in the static environment (6 obstacles).

Algorithm name	Path length (m)
MOEPSO	**14.4743**
Hybrid PSO-MFB [12]	14.6384
Standard ABC [42]	14.8821
DABC [40]	14.7422
MAABC [40]	14.7163

**Fig 30:**
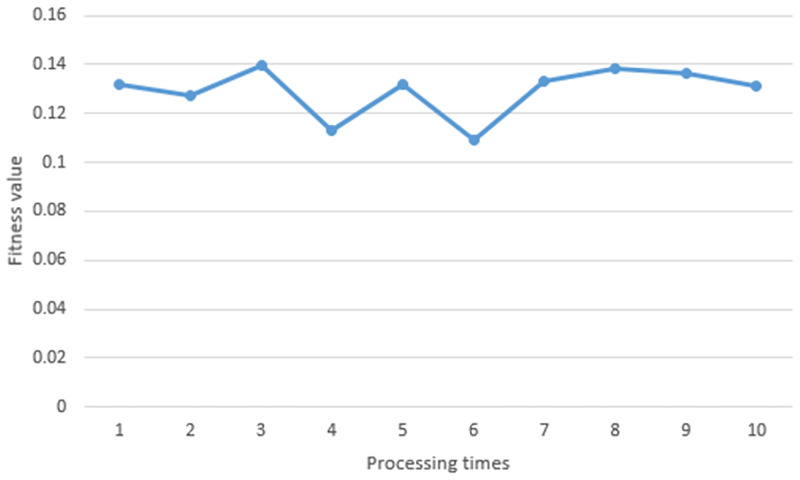
The fitness value obtained in a static environment (6 obstacles).

**Fig 31:**
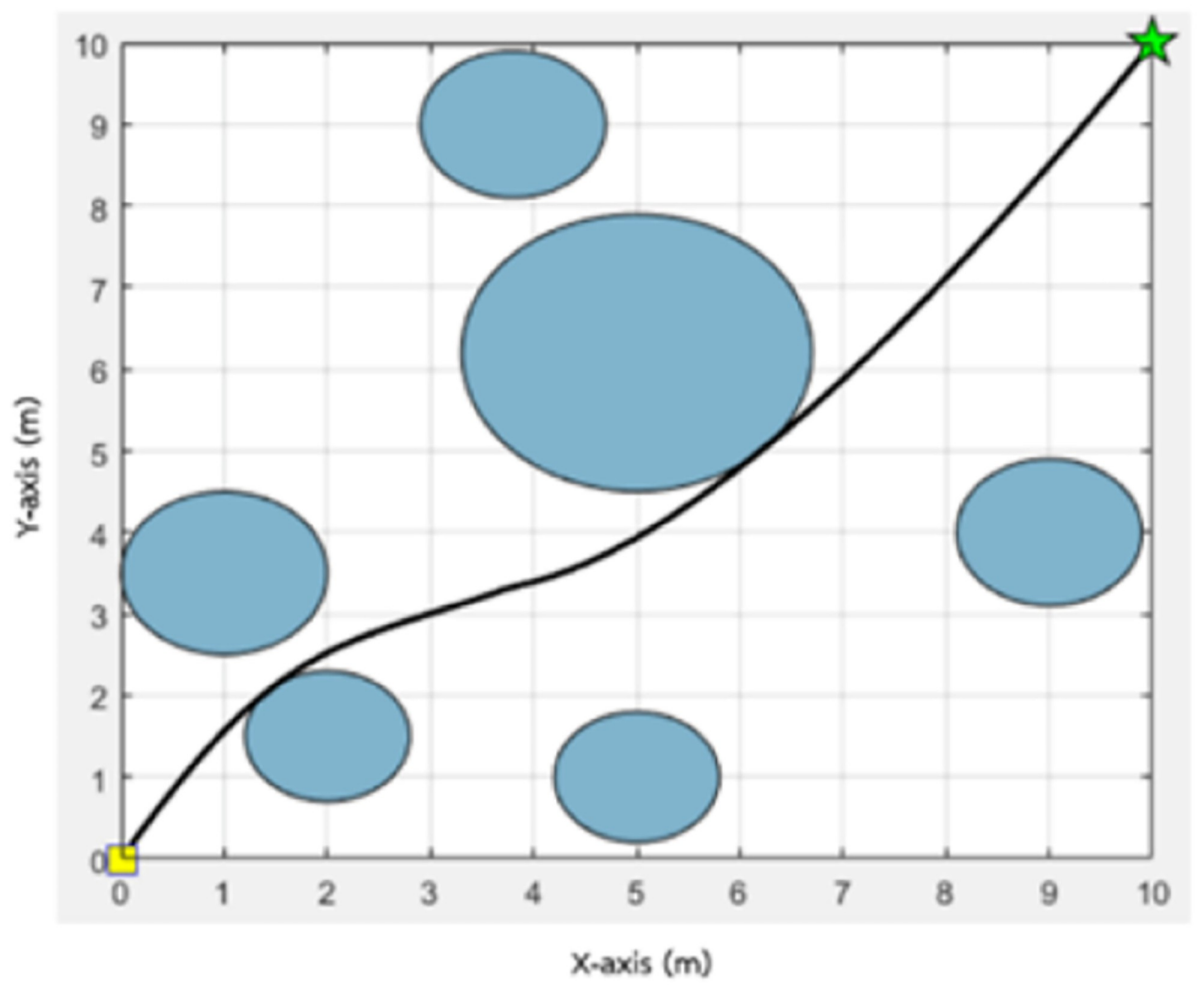
The path obtained from the MOEPSO algorithm in a static environment (6 obstacles).

Experimenting in a static environment with 6 obstacles, we ran the proposed algorithm 10 times. Fig 30 shows the fitness value obtained from each processing time. At the first time, the fitness value was 0.1316. At the second time, the fitness value was 0.1271. At the third time, the fitness value was 0.1395. At the fourth time, the fitness value was 0.1128. At the fifth time, the fitness value was 0.1319. At the sixth time, the fitness value was 0.1095. At the seventh time, the fitness value was 0.1333. At the eighth time, the fitness value was 0.1381. At the ninth time, the fitness value was 0.1362. At the last time, the fitness value was 0.1312. In each iteration, the particles with the best fitness value were chosen as the waypoint of path. These points are drawn as the optimal path as shown in Fig 31.

**Fig 32:**
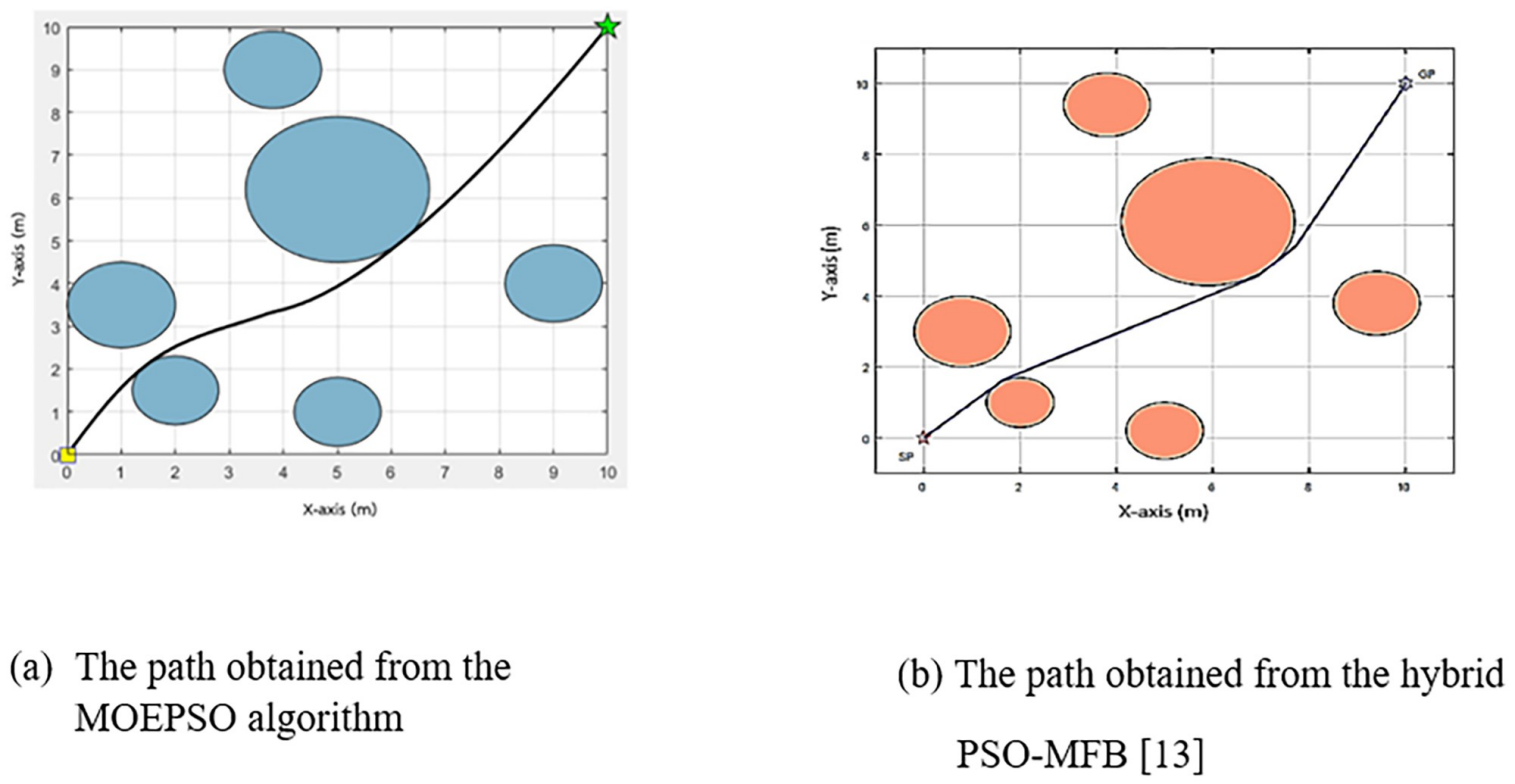
A comparison of the paths obtained from the MOEPSO and the hybrid PSO-MFB [12].

Fig 32 shows a comparison of the paths obtained from the path planning experiment in a static environment with 6 obstacles. Fig 32(a) shows the path obtained from the MOEPSO algorithm, and Fig 32(b) shows the path obtained from the hybrid PSO-MFB algorithm. It can be seen that the path obtained from the MOEPSO algorithm has a smaller distance value from the starting point to the destination point than the path obtained from the hybrid PSO-MFB algorithm.

### The result of path planning in dynamic environment

**Fig 33:**
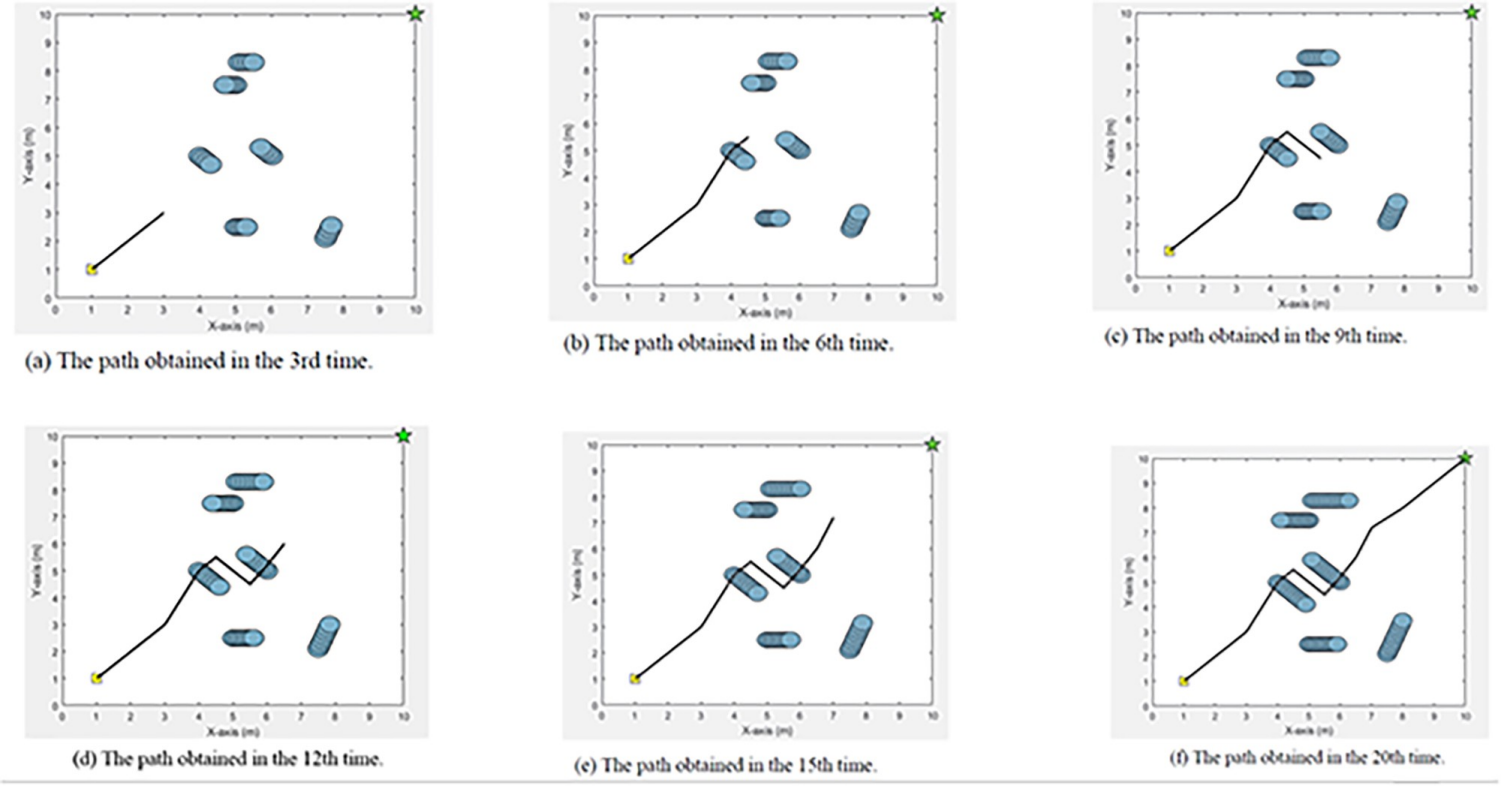
The path obtained from the MOEPSO algorithm in a dynamic environment.

For the path planning experiment in a dynamic environment, the proposed algorithm was ran 20 times. At each time, obstacles will change their positions continually. The experimental results of path planning in a dynamic environment are shown in Fig 33.

Fig 33 shows the path obtained from the MOEPSO algorithm in path planning with a dynamic environment where obstacles can move. The starting point was determined at position (1,1), and the destination was determined at position (10,10). In such an environment, there were obstacles moving in straight lines and nonstraight lines. Their characteristics are shown in Tables 5 and 6. The robot moves from the starting point to the destination point along the path obtained from the proposed algorithm. When obstacles are close to the robot, the installed sensor scans the positions and directions of obstacles. The proposed algorithm uses these data to adjust a new path for the robot to avoid obstacle collision. The robot can move continuously until it reaches the destination. The shortest path obtained from the proposed algorithm was 12.2381 meters, and the average time spent on processing was 0.2968 minutes.

**Fig 34:**
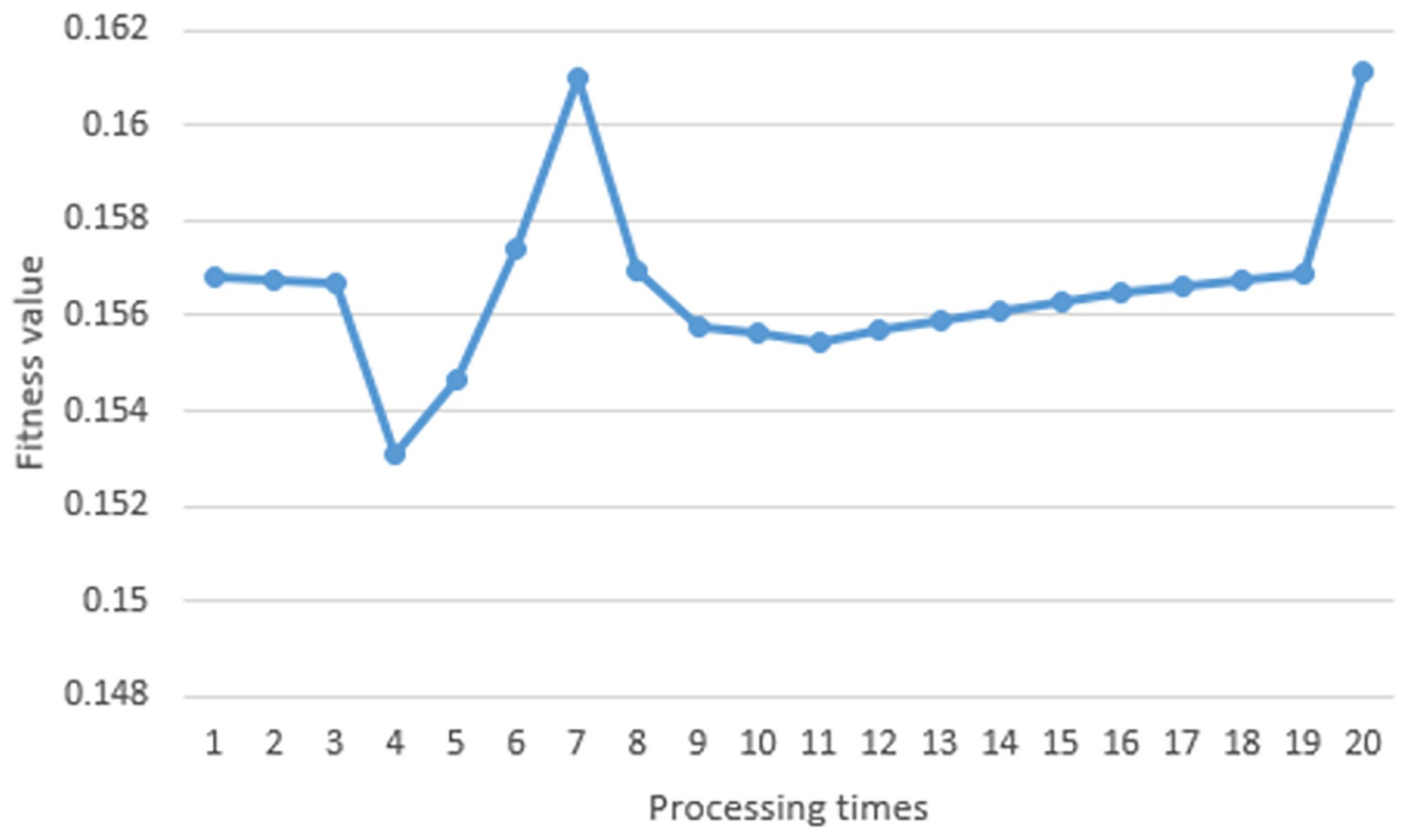
The fitness value obtained in a dynamic environment.

Experimenting in a dynamic environment, we ran the proposed algorithm 20 times. Fig 34 shows the fitness value obtained from each processing time. At the first time, the fitness value was 0.1568. At the second time, the fitness value was 0.1668. At the third time, the fitness value was 0.1567. At the fourth time, the fitness value was 0.1531. At the fifth time, the fitness value was 0.1547. At the sixth time, the fitness value was 0.1574. At the seventh time, the fitness value was 0.161. At the eighth time, the fitness value was 0.1569. At the ninth time, the fitness value was 0.1558. At the tenth time, the fitness value was 0.1556. At the eleventh time, the fitness value was 0.1554. At the twelveth time, the fitness value was 0.1557. At the thirteenth time, the fitness value was 0.1559. At the fourteenth time, the fitness value was 0.1561. At the fifteenth time, the fitness value was 0.1563. At the sixteenth time, the fitness value was 0.1565. At the seventeenth time, the fitness value was 0.1566. At the eighteenth time, the fitness value was 0.1568. At the nineteenth time, the fitness value was 0.1569. At the last time, the fitness value was 0.1611. In each iteration, the particles with the best fitness value are chosen as the waypoint of path. These points are drawn as the optimal path as shown in Fig 33.

Based on the path planning tests in the static environment and dynamic environment, it can be seen that the proposed algorithm can create paths from the starting point to the destination with shorter paths than other algorithms used to compare operational performance. In this regard, the operations of the MOEPSO algorithm require generating a group of initial populations by randomly selecting various values from the search space to determine feasible waypoints, giving the algorithm sufficient choices to be selected as waypoints having characteristics that meet the criteria: the shortest path, the smoothest path and the safest path. In addition, the proposed algorithm improves the operations of particles using evolutionary operators such as mutation, crossover, and selection, which increase particle strength at each iteration, resulting in particles with better fitness values being used as parents for generating particles in the next generation. In addition, the improvement of the equations in terms of weighted value adjustment in the movement of particles at each iteration gives rise to each particle being able to find a wide range of suitable answers (global search).

With regard to path smoothness, the proposed algorithm can create smoother paths compared to other algorithms. The reason is that the particles generated from the proposed algorithm contain values of all possible real numbers in the search space. The real number values from those particles are very detailed. Therefore, when they are used to connect to each waypoint, very smooth paths can be obtained.

In terms of path safety, the proposed algorithm can create a path with the farthest distance from obstacles according to the objective because the sensor installed in the robot can identify the positions of each obstacle quite precisely, including a large number of feasible waypoints at each iteration that the algorithm can use to adjust the path for obstacle avoidance.

In addition, the proposed algorithm requires the least time processing in all environments compared to other algorithms in previous studies. The reason is that the proposed algorithm does not work in combination with other algorithms, but all components work under the operation of a single algorithm. Moreover, feasible waypoints within the radius of obstacles or blocked by obstacles are handled using a simple random method, resulting in time savings in processing.

The proposed algorithm still has limitations in the crossover process. At this stage The crossover operator is used to generate the trial particles from each particle and its mutant particle. The trial particle will be created by Eq (12). From the equation it can be seen that the value of the trial particle depends on the value of b being the dimension of the problem. The small dimension problem results in a lack of diversity of crossing over. This leads to being trapped in a local optimal too early. On the other hand, the large dimension problem will increase the chances for a more diverse selection of mutants or initial particle to crossing over. This leads to the development of answers with better suitability.

## Conclusions and future works

This article proposes a new path planning algorithm for autonomous mobile robots. The algorithm is developed from evolutionary particle swarm optimization (EPSO). It is called multi-objective evolutionary particle swarm optimization (MOEPSO). In this study, the proposed algorithm is used to solve path planning problems for autonomous mobile robots by considering path length, smoothness, and safety. In the procedures of creating feasible waypoints, each particle has an increased ability to find suitable answers using evolutionary operators, i.e., mutation, crossover and selection. Furthermore, the equations of weighted value adjustment in the movement of particles in each iteration are improved to ensure that the best fit paths are obtained. Feasible waypoint handling is proposed when these points are within the radius of obstacles or blocked by obstacles. In terms of obstacle detection, the robot can perceive the surrounding environment using a single sensor. The sensor works by rotating around itself in a clockwise direction and emitting a sensor light to strike obstacles. The distance between the robot and the obstacles is calculated from the duration that the light travels from the emitter and reflects back to the receiver following the time of flight (TOF). In addition, it can identify the positions of obstacles according to the left-hand rule of the coordinate system.

The proposed algorithm is tested in both a static environment and dynamic environment with different models. The size of the robot is taken into consideration, as well as the size of the radius of the robot and the radius of the obstacles. Based on the test results, it can be said that the MOEPSO algorithm finds optimal paths better than other algorithms in terms of path length, smoothness, and safety. In addition, it takes less processing time than other algorithms.

In this article, the shape of the static obstacle is the only spherical shape. Therefore, other shapes of the static obstacles should be simulated as well. In the future work, we are interested in studying path planning with various shapes of the static obstacles. In addition, the dynamic environment is not diverse. In the future work, we are interested in testing the proposed algorithms with various dynamic environments. Moreover, the path planning of multiple robots is in our plan.
